# Emerging priorities and concerns in the wake of the COVID-19 pandemic: qualitative and quantitative findings from a United States national survey

**DOI:** 10.3389/fpubh.2024.1365657

**Published:** 2024-06-19

**Authors:** Carolyn E. Schwartz, Katrina Borowiec, Ariel H. Waldman, Tai Sutherland, Briana Contreras, Elizabeth Abatan, I-Chan Huang, Gudrun Rohde, Bruce D. Rapkin, Richard L. Skolasky

**Affiliations:** ^1^DeltaQuest Foundation, Inc., Concord, MA, United States; ^2^Departments of Medicine and Orthopaedic Surgery, Tufts University Medical School, Boston, MA, United States; ^3^Department of Measurement, Evaluation, Statistics, and Assessment, Boston College Lynch School of Education and Human Development, Chestnut Hill, MA, United States; ^4^Department of Orthopaedic Surgery, Johns Hopkins University School of Medicine, Baltimore, MD, United States; ^5^Department of Epidemiology and Cancer Control, St. Jude Children’s Research Hospital, Memphis, TN, United States; ^6^Faculty of Health and Sport Sciences at University of Agder and Department of Clinical Research Sorlandet Hospital, Kristiansand, Norway; ^7^Department of Epidemiology and Population Health, Albert Einstein College of Medicine, New York, NY, United States

**Keywords:** response shift, COVID-19, quality of life, meaning, priorities, values, health, interpersonal

## Abstract

**Purpose:**

The present study examines how the coronavirus disease 2019 (COVID-19) experience affected values and priorities.

**Methods:**

This cross-sectional study collected data between January and April 2023, from 1,197 individuals who are chronically ill or part of a general population sample. Using open-ended prompts and closed-ended questions, we investigated individuals’ perceptions about COVID-19-induced changes in what quality of life means to them, what and who are important, life focus, and changes in norms and stressors. Data analyses included content and psychometric analysis, leading to latent profile analysis (LPA) to characterize distinct groups, and analysis of variance and chi-squared to compare profile groups’ demographic characteristics.

**Results:**

About 75% of the study sample noted changes in values and/or priorities, particularly in the greater prominence of family and friends. LPA yielded a four-profile model that fit the data well. Profile 1 (Index group; 64% of the sample) had relatively average scores on all indicators. Profile 2 (COVID-Specific Health & Resignation to Isolation Attributable to COVID-19; 5%) represented COVID-19-specific preventive health behaviors along with noting the requisite isolation and disengagement entailed in the social distancing necessary for COVID-19 prevention. Profile 3 (High Stress, Low Trust; 25%) represented high multi-domain stress, with the most elevated scores both on focusing on being true to themselves and perceiving people to be increasingly uncivil. Profile 4 (Active in the World, Low Trust; 6%) was focused on returning to work and finding greater meaning in their activities. These groups differed on race, marital status, difficulty paying bills, employment status, number of times they reported having had COVID-19, number of COVID-19 boosters received, whether they had Long COVID, age, BMI, and number of comorbidities.

**Conclusion:**

Three years after the beginning of the worldwide COVID-19 pandemic, its subjective impact is notable on most study participants’ conceptualization of quality of life, priorities, perspectives on social norms, and perceived stressors. The four profile groups reflected distinct ways of dealing with the long-term effects of COVID-19.

## Introduction

It is generally acknowledged that societal stressors impact the immediate health-related quality of life (QOL) and well-being of affected individuals, and recent research has documented impacts on longer-term outcomes. For example, the extreme economic challenges of the Great Depression led to immediate health effects due to food and housing insecurity, and extreme mental health problems including anxiety, depression, and even suicidality (1, 2). Longer-term impacts reflected life-long Depression-induced values and priorities, such as hoarding (3), distrust of financial institutions (4), and extreme frugality (5). Accelerated age-related physiological damage among surviving cohort members has also been reported (6), as well as faster epigenetic aging among children who were *in utero* during the Great Depression (7). Other examples of societal stressors that had large, short- and long-term effects on QOL and broader psychosocial outcomes include natural disasters, such as Hurricane Katrina (8) or the California wildfires (9), human-induced suffering, such as the Holocaust (10), 9/11 (11), and mass shootings (12), and infectious-disease pandemics, such as the 1918 Spanish flu (13), the avian flu (14), and human immunodeficiency virus/acquired immunodeficiency syndrome (HIV/AIDS) (15).

The coronavirus disease 2019 (COVID-19) pandemic has some similarities to these earlier crises but is distinct in several important ways. Similar to the 1918 Spanish flu, COVID-19 was strikingly global, fast-moving, and lethal (16), although the mortality attributed to the Spanish flu was 50–100 million deaths globally and 675,000 in the United States over 10 months (0.64% of the total population), whereas the mortality attributed to COVID-19 is currently estimated to be 15–16 million people worldwide in the two years of 2020 and 2021 [0.02% of the total population (17–21)]. However, whereas the Spanish flu virus disappeared before an effective vaccine could be developed (22), the coronavirus continued its devastation for a full year until effective vaccines became available (23, 24), and these vaccines continued to evolve as the virus mutated. By early 2023, many people around the world were vaccinated and the death toll was greatly reduced to about 30% of the 2022 rate in Western countries (25–27). Thus, vaccine availability differentiated COVID-19 from this earlier pandemic and transformed the viral illness from primarily deadly to a potentially disabling but less frequently fatal condition.

In the United States, a rapidly changing understanding of the coronavirus as well as misinformation transmitted from political (28, 29) and public-health leaders (30) and the media (31, 32) had great costs (33). Instead of a clear message about best practices for self-protection and risk factors for transmission, such information became politicized (34). Social distancing was instituted, a measure that had both benefits in reducing exposure but also costs such as adverse effects on mental health, glycemic control in diabetes, and other health problems (35, 36). Social distancing became an emblem of what political message an individual believed, rather than a clear directive to all in the interest of the greater good (28, 32, 37). Social upheaval, social divisiveness, and mistrust were part of the experience of the pandemic, fueled by social media (38).

The initial trauma of COVID-19 was documented in global research done on the deeply distressing and disturbing experience early in the pandemic (39–41). This research complemented the growing and substantial evidence base related to the clinical science of viral transmission and containment (42–45). It has documented beyond doubt the lasting imprint of sociodemographic and racial inequities on the experience of COVID-19, exacerbating the physical, psychological and social impacts of COVID-19 among people of color (46–48).

A small subset of this early research also noted important changes and changeability in values and priorities. For example, researchers in Australia documented that “conservation values” that emphasized order and stability became more important early in the pandemic, but these same values became less important by late 2020 (49). While COVID-19-driven “cocooning” led to reduced reported enjoyment and increased loneliness among older adults in Ireland, their priorities shifted to concern about “protecting the development of children” and “enjoying life as much as possible (50).” In the United Kingdom, taking responsibility and being concerned about security were core values among those compliant with COVID-19 behavioral guidelines, and perceiving that others shared these values elicited a sense of connectedness to them (51). Similarly, in Poland, people reported increased valuing of self-direction, security, conformity, humility, and caring, and a reduced emphasis on hedonism early in the lockdown period of the pandemic (52). In the state of Vermont, United States, people reported a growing connection with nature and its value in helping them cope, inspiring them, and providing access to enjoyable activities despite COVID-19-imposed limitations (53).

In addition to early changes in values and priorities, researchers noted changes in social norms early in the pandemic. In Spain, individuals most closely connected with a community were more willing to sacrifice for others, and this altruism was motivated more by social norms than by a perceived threat (54). Social norms were also found to drive preventive health behaviors in Germany and Japan (55) and in a global study of 115 countries (56).

There is, however, a paucity of research on changes in values, priorities, and social norms at this later stage of the pandemic, when COVID-19 is entrenched in our collective reality. COVID-19 is no longer treated as a public health crisis by leaders. The current public health protocol is routine booster vaccines and vigilance to the onset of symptoms. For many, when infected with COVID-19, it resembles a normal flu, not a life-threatening event. For others, however, an initial COVID-19 infection may lead to Long COVID (57), a multisystemic condition impacting multiple organ systems. At this later stage, the memory of social-distancing and preventive health behaviors is still relatively fresh, but life has largely resumed to pre-COVID-19 normal for many people. However, recent U.S. polling data from November 2023 suggests that about 50% of adults are taking at least one or more of the following precautions: avoiding large gatherings, avoiding travel, avoiding dining indoors at restaurants, wearing masks in crowds, and testing for COVID-19 before visiting with family and friends (58). Notably, only 39% of White participants reported taking precautions compared to 72% of Black and 68% of Hispanic participants. It is also important to note that this push to “return to normal” has in many respects further marginalized disabled and immunocompromised people (59).

In sum, the experiences of the pandemic may have had broad ramifications, for example in people’s trust in science and public health, social activity and willingness to affiliate, and sense of equity. The present study thus aimed to understand how the COVID-19 experience led to perceived changes in values, priorities, social norms, and stressors. Using methods that relied on data using both open-ended prompts and closed-ended questions, it utilized thematic analysis, data-reduction tools, and mixed-methods analyses to investigate individuals’ perceptions about COVID-19-induced changes in what QOL means to them, about what is important, who is important, and what they should focus their life energy on.

## Materials and methods

### Sample and design

This study utilized cross-sectional data from the fourth and final data collection of a quasi-experimental, longitudinal study of the psychosocial impact of the COVID-19 pandemic. The data were collected via an online structured questionnaire between January 19 and April 12, 2023. Study participants were recruited via Rare Patient Voice^1^ and Ipsos Insight^2^ to yield a general-population sample of United States adults who were heterogeneous in terms of health and nationally representative in terms of age distribution, gender, region, and income. Both Rare Patient Voice and Ipsos Insight are for-profit, panel-research organizations that facilitate study recruitment by emailing their panel members, confirming compliance with study eligibility criteria, and providing links to the baseline survey outreach done by DeltaQuest Foundation, a not-for-profit medical research organization. Participants were not paid monetarily for their participation.

Criteria for eligibility were age 18 or older, able to complete an online questionnaire, and able to provide informed consent. The survey was administered through the secure Alchemer engine,^3^ which is compliant with the United States Health Insurance Portability and Accountability Act. Alchemer is a for-profit organization. The protocol was reviewed and approved by the WCG Independent Review Board (#2021164), and all participants provided informed consent prior to beginning the survey.

### Measures

Perceived changes in values and priorities were assessed using four open-ended questions regarding participants’ appraisal of changes in perspective and seven closed-ended questions on changes in priorities, seven on perceived changes in social norms, and 22 life-stress items adapted from the Urban Life Stressors Scale (60, 61). The open-ended questions asked: “When you think back on your experiences with the COVID pandemic, how have you changed how you think about… (1) the meaning of quality of life; (2) what is important in your life; (3) who is important in your life; and (4) what you want to focus on or spend your life energy on?” The closed-ended questions on changes in priorities over the COVID-19 pandemic queried job (3 items), relationships (2 items), and having alone (1 item) or free (1 item) time. The social-norms questions queried confidence in public health strategies for preventing the spread of COVID-19 (e.g., mask-wearing, vaccination), incivility (e.g., impoliteness, anger), leadership/media propriety (e.g., truth-telling, protecting best interests of the general public and vulnerable populations). One additional item “the COVID-19 pandemic” was added to the usual items from the Urban Life Stressors Scale for the current study. Supplementary Text provides the full text of these open-and closed-ended questions.

#### Demographic characteristics

Demographic characteristics included age, gender, with whom they live, cohabitation/marital status, race, ethnicity, education, region, height and weight [to compute body mass index (BMI)], reported difficulty paying bills, employment status, smoking status, years since chronic illness/comorbidity diagnosis if applicable, number of comorbidities, whether/how many times the individual had COVID-19, COVID-19 vaccination history, whether they believed they had Long COVID, and whether they received assistance completing the survey.

### Data analysis

Descriptive statistics were used to describe the study sample characteristics.

#### Qualitative analysis

The open-ended data were coded into themes by six trained raters (CS, AW, TS, BC, EA, and RS), according to an existing framework from two decades of appraisal research (62). This existing framework provided a standardized protocol and comprehensive codebook originally derived using both deductive and inductive approaches in an extensive sorting procedure (63). An initial review of the open-text responses to the four prompts led us to utilize themes from past work on QOL Meaning for the QOL Meaning prompt, and on Goal Delineation themes for the Who, What, and Focus prompts. From this starting point, themes were iteratively refined based on emergent themes in the data, yielding a set of 22 themes used for the QOL Meaning prompt, and 55 themes for the Who, What, and Focus prompts. Themes in the current data were coded as “1” or “0” depending on whether they were reflected or not, respectively, in the individual’s written text. For each prompt, a theme of “No Direct Answer” was used if the respondent did not provide an answer (i.e., left blank) or answered a different question than the one that was asked. For example, in response to the question “…how have you changed how you think about the meaning of QOL?,” a non-blank No Direct Answer was “My quality of life is ok” or “Yes.”

Each text entry could be coded for as many themes as were reflected in the set for the corresponding prompt. Therefore, one entry could elicit one theme or more than one depending on its wording. For example, in response to the What’s Important prompt, one individual had written “I have tried to slow down more and not focus on work so much. I also realized how much I enjoyed my time outdoors and take more intentional walks,” which was coded as reflecting Creating Moments & Memories, Epiphanic Clarity, Health & Wellness, Prioritization, and Work & Unemployment. In contrast, another individual’s “What’s Important” response was “Cannot take things for granted” which was coded with the single theme of Epiphanic Clarity. Responses stating that there was no change in a particular perspective were coded as “No Change.”

Training took place in four 1.5 h sessions to understand the protocol and to utilize fully and expand as needed the codebook. Raters coded an initial set of 10 participants’ data (from all four prompts), followed by a discussion of differences across raters. Incorporating exchanged feedback, they then coded the next 10 participants’ data (again all prompts) for three more rounds, at which point comparison and discussion revealed almost no differences across raters. Raters coded data from 41 responses (all four prompts), from which inter-rater reliability per prompt was computed in two ways on the 246 test responses (6 raters * 41 participant entries).

#### Inter-rater reliability

Fleiss’s kappa (64) assessed the degree of agreement over and above what would be expected by chance. This variant on the more familiar Cohen’s kappa (65) is used in cases of more than two raters. While there are no generally accepted guidelines for a desirable level of either form of kappa, some healthcare researchers have proposed values from 0.41–0.60 as “moderate,” 0.61–0.80 as “good,” and 0.81–1.00 as “very good” (66, 67). Once the reliability analysis suggested sufficient consistency across raters to proceed, the remaining entries were randomly divided among raters to complete the coding of the open-text data.

#### Selection bias

To address possible selection biases associated with remaining in the longitudinal sample versus being lost to follow-up from the study baseline, chi-squared tests of independence or analysis of variance (ANOVA) tests were computed comparing the retention and attrition samples (i.e., the current study sample at this final follow-up versus the sample who participated in the study at baseline but not in this fourth and final follow-up) on their demographic, characteristics at baseline. Cohen’s criteria (68) for small, medium and large effect size facilitated interpretation of results.

#### Data reduction

To reduce the number of variables used in subsequent inferential analyses, exploratory principal axis factor analysis with varimax rotation was used on the closed-ended questions, separately for Changes in Priorities, Social Norms, and Life Stress. Extracted factors had to have eigenvalues greater than 1.0, and a cut-point of 0.50 was used for including an item in a factor score, corresponding to medium loadings (69). Internal consistency reliability on the extracted factors was computed using Cronbach’s alpha reliability coefficient. Methodologists generally recommend a minimum internal constancy reliability [i.e., alpha (*α*) reliability] coefficient between 0.65 and 0.8, and *α* coefficients below 0.5 are generally considered unacceptable (70, 71). Using only themes with at least 25 participant endorsements within a prompt (i.e., 2% of the sample), principal components analysis (PCA) with varimax rotation was used to reduce the number of variables into composite scores for the coded open-text data, separately by prompt (i.e., QOL Meaning, What’s Important, Who’s Important, and Focus On). The “No Direct Answer” or “No Change” themes were excluded from the PCA. Extracted components had to have eigenvalues greater than 1.0. These two data-reduction techniques reduced the number of variables from 34 closed-ended items to 9 factors, and 46 distinct themes to 21 composites (30 in total), of which one did not load on any profile and thus was not retained in the variables used in the final Latent Profile Analysis (LPA) solution (29 in total). The 29 factor and composite scores were transformed to be on a *T*-score metric, with a mean of 50 and standard deviation of 10, for ease of comparability and interpretability.

LPA (72) is a person-centered method, rather than a variable-centered method. Accordingly, LPA was then used to identify subsets of persons with shared characteristics (i.e., response shift effects) using the 29 indicators with sufficient prevalence. We tested models of one through five profiles and selected the best fitting model based on the lowest Akaike information criterion (AIC) and Bayesian information criterion (BIC) statistics, the highest entropy statistic, and Lo Mendell Rubin adjusted likelihood ratio test (LRT) results. Mplus was used to estimate the most likely profile for each person.

#### Inferential analysis

Given the high classification accuracy (i.e., entropy) of the final LPA model, we then used the resulting profiles to examine bivariate relationships between the profiles and the set of demographic variables described above, using chi-squared analyses for categorical variables and univariate ANOVA models for continuous variables. Rather than relying on *p*-values, which would have been unduly affected by the relatively large sample size of the present study, we focused on effect sizes (ES) to facilitate interpretation, using Cohen’s cut-offs for explained variance (eta^2^) (68). Table values were conditionally formatted to highlight the small, medium, and large ES of the magnitude of eta^2^ estimates (i.e., 0.01, 0.06, and 0.14, respectively). More saturation reflects larger ES.

Statistical analyses were implemented using IBM SPSS version 29 (73), Mplus version 8.8 (74), and Microsoft Excel.

## Results

### Sample

The study sample included 1,197 individuals. This sample reflects 25.3% of the baseline sample (*n* = 4,757), 69.1% of the follow-up 1 sample (*n* = 1734), and 95.5% of the follow-up 2 sample (*n* = 1,255). The participation rate of the baseline sample is unknown because the number of people to whom the panel research companies invited to participate is unknown. Table 1 provides the sociodemographic characteristics of the overall study sample. Compared to those who were lost to follow-up from the baseline data set of 4,757 individuals, the 1,197 retained study participants were less likely to report difficulty paying bills, were more likely to report having a college or postgraduate degree and were older (all small effect sizes; see Supplementary Table S1).

**Table 1:** Overall sample demographic characteristics (*n* = 1,197).

Variable	Category	#	%
Role	Patient	791	66%
	Caregiver	172	14%
	Both	47	4%
	Neither	187	16%
	Missing	0	0%
Gender	Male	190	16%
	Female	1,001	84%
	Other	6	1%
	Prefer not to answer	0	0%
Race	White	1,087	91%
	Person of color/multiracial	81	7%
	Prefer not to answer	29	2%
Living alone	Yes, living alone	182	15%
Marital status	Never married	159	13%
	Married	701	59%
	Cohabitation/Domestic	65	5%
	Separated	15	1%
	Divorced	158	13%
	Widowed	94	8%
	Prefer not to answer	5	0%
Difficulty paying bills	Not at all difficult	673	56%
	Slightly difficult	255	21%
	Moderately difficult	131	11%
	Very difficult	71	6%
	Extremely difficult	50	4%
	Not applicable/Prefer not to answer	17	1%
Employment status	Employed	480	40%
	Unemployed	85	7%
	Retired	361	30%
	Medically disabled	256	21%
	Do not know/Prefer not to answer	15	1%
Education (at baseline)	Less than high school graduate	6	1%
	High school diploma/GED	97	8%
	Trade or technical degree	76	6%
	Some college	266	22%
	College degree	374	31%
	Postgraduate degree	375	31%
	Missing	3	0%
Region (at baseline)	East North Central	179	15.0
	East South Central	48	4.0
	Middle Atlantic	135	11.3
	Mountain	111	9.3
	New England	67	5.6
	Other US or International	76	6.3
	Pacific	189	15.8
	South Atlantic	266	22.2
	West North Central	59	4.9
	West South Central	67	5.6
Currently smoke or vape	Not at all	1,076	90%
	Some days	37	3%
	Every day	76	6%
	Prefer not to answer	8	1%
Received help completing survey	Yes	13	1%
Number of times has had COVID-19	0	519	43%
	1	482	40%
	2	133	11%
	3	32	3%
	4	6	1%
	5	2	0%
	Other	14	1%
	Do not remember	6	1%
	Missing	3	0%
Received a COVID-19 vaccine	No	114	10%
	Yes	1,066	89%
	Do not remember	5	0%
	Missing	12	1%
Received 1+ COVID-19 booster	No	218	18%
	Yes	948	79%
	Do not remember	2	0%
	Missing	29	2%
Number of COVID-19 boosters (if received 1 or more boosters)	1	142	12%
	2	284	24%
	3	305	25%
	Other	62	5%
	Do not remember	2	0%
	Missing	153	13%
Have Long COVID	Definitely not	601	50%
	Probably not	214	18%
	Probably yes	100	8%
	Definitely yes	59	5%
	Do not know	116	10%
	Missing	107	9%
		**Mn**	**SD**
Age		57.13	13.42
	Missing	0	
Body mass index (at baseline)		29.50	7.80
	Missing	39	
Comorbidities		4.08	2.57
	Missing	3	
Time since diagnosis (diagnosis date reported at baseline)		18.00	12.58
	Missing	42	

GED, General Educational Development (i.e., high-school equivalency test); SD, standard deviation; Mn, mean; SD, standard deviation.

### Reliability of open-text coding

Supplementary Table S2 provides a full listing of the coding themes for the open-text prompts as well as definitions and examples of each. Inter-rater reliability analyses demonstrated good reliability for the prompts related to QOL meaning, What’s Important, and Who’s Important, and moderate reliability for the Focus On Prompt (average kappa = 0.652, 0.621, 0.651, and 0.544, respectively; Supplementary Table S3).

### Prevalence of coded themes

**Figure 1:**
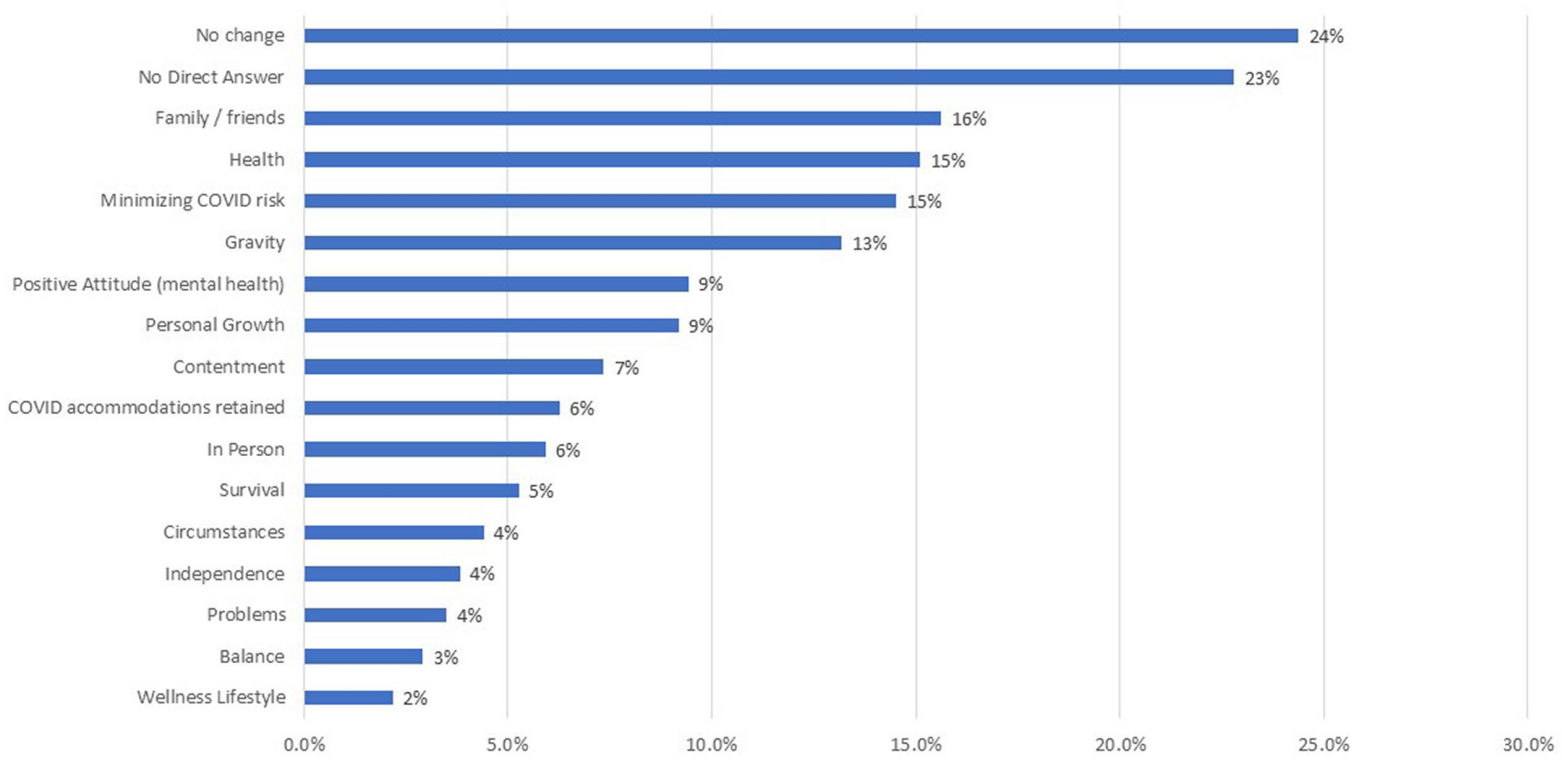
Prevalence of themes for QOL meaning prompt. This bar chart shows the QOL meaning themes, ranked by prevalence of endorsement. Only those themes retained for subsequent analysis (i.e., >2% endorsement) are shown.

Supplementary Table S4 provides information about the prevalence of endorsement of each of the coded themes. In order to be included in subsequent analysis, a theme had to be endorsed by at least 25 individuals, representing 2% of the sample. Figure 1 shows the ranked prevalence of QOL Meaning themes for those themes retained for subsequent analysis. Almost one-quarter of the sample endorsed “No Change” and 23% provided no direct answer to how their thinking about QOL changed as a result of the COVID-19 pandemic. Among the remaining individuals who commented on specific aspects of changes in QOL meaning, the most prevalent themes related to family/friend, health, minimizing COVID-19 risk, and gravity (i.e., pandemic-induced renewed appreciation for something). Supplementary Figure S1 provides a full listing of all QOL Meaning themes and their prevalence.

**Figure 2:**
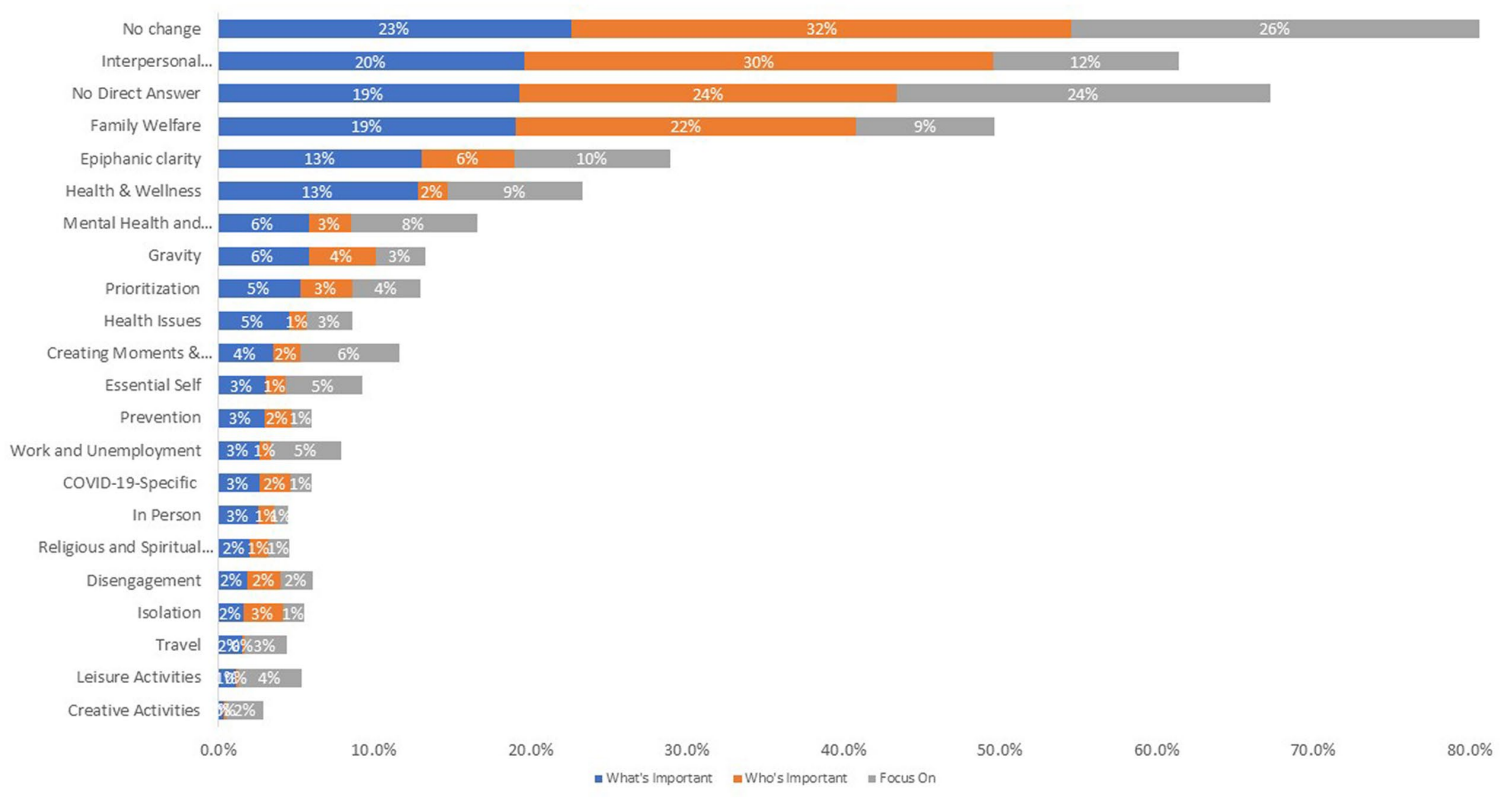
Prevalence of themes for what is important, who is important, and life-energy focus prompts. This stacked bar chart shows the themes coded for what is important, who is important, and life-energy focus prompts, ranked by prevalence of endorsement. Only those themes retained for subsequent analysis (i.e., >2% endorsement) are shown.

Figure 2 shows a stacked bar chart illustrating the ranked prevalence of the What’s Important, Who’s Important, and Life-Energy Focus prompts for those themes retained for subsequent % analysis. Again, about one quarter of the sample endorsed “No Change” on these prompts, and slightly fewer provided no direct answer. Among the remaining individuals who commented on specific aspects of changes in priorities, the most prevalent themes related to interpersonal relationships, family welfare, epiphanic clarity (i.e., relating to a moment where suddenly realize something as important), and health & wellness. Supplementary Figure S2 provides a stacked bar showing a full listing of all themes used to code What, Who and Life-Energy Focus prompts and their prevalence.

### Data reduction

Factor analyses reduced the 34 items to nine factors that explained substantial variance and generally demonstrated acceptable internal consistency reliability. (See Supplementary Table S5 for details.)

Factor analyses on Perspective Changes items yielded two factors that explained 53% of the variance. “*Inner Life & Relationships*” included items focusing on having unscheduled time and improving relationships (Cronbach’s *α* = 0.79). “*Job*” included items reflecting the importance of one’s job and the organizational context (Cronbach’s *α* = 0.72).

Social Norms items were summarized by three factors that explained 52% of the variance (*α* = 0.73). “*Public Health Confidence & Consideration*” included items related to confidence that others would follow public-health standards for preventing the spread of disease (*α* = 0.75). “*Trust in Leaders & Media*” included items related to trust that leaders have the best interest of the general public when making COVID-19-policy decisions and that the media provides accurate information about COVID-19 (*α* = 0.64). “*Public Incivility*” included items related to public displays of impoliteness, inconsideration, and anger.

Stress items were summarized by four factors that explained 43% of the variance. “*Health-Related QOL Stress*” included items related to the usual domains of health-related QOL (i.e., physical health, mental health, social functioning) as well as access to medical care and public services, and COVID-19-pandemic related stress (*α* = 0.84). “*Systemic racism/inequity*” included items related to inter-racial relations, interactions with police, and experiences with racism and crime (*α* = 0.75). “*Financial Hardship*” included items related to financial, housing or job/unemployment concerns (*α* = 0.74). “*Family Relationship Stress*” included items related to stress raising children and with a marital/romantic relationship (*α* = 0.58). The latter factor’s internal consistency reliability was lower than the usual acceptable norms but higher than what would be considered unacceptable.

PCA’s were done only on themes with at least 25 endorsements per prompt (i.e., 2% of the sample) in order to yield a robust solution. Of the 23 QOL Meaning coding themes, 14 were retained for the analysis. Of the 56 coding themes used for the What’s Important, Who’s Important, and Focus On prompts, 14, 8, and 11 were retained, respectively. (See Supplementary Table S6 for details.)

QOL Meaning was summarized by six composite scores that explained 56% of the variance. “*Surviving COVID-19*” reflected themes related to minimizing COVID-19 risk, retaining COVID-19 prevention behaviors, and survival. “*Post-traumatic Growth*” reflected themes related to personal growth, positive attitude, and balance. “*Interpersonal Connections*” reflected themes related to valuing family / friends and in-person interactions. “*Renewed Appreciation*” reflected themes related to taking life more seriously, appreciating the small things in life, and experiencing a sense of gratitude. “*Health Concerns*” reflected themes related to short-term specific problems and health issues. “*Circumstances*” reflected themes downplaying wellness and emphasizing longer-term situations of concern.

What’s Important was summarized by six composite scores that explained 54% of the variance. “*COVID-19 Prevention*” reflected themes related to COVID-19-specific prevention. “*Primacy of Employment*” reflected themes related to prioritizing work. “*Interpersonal Connections*” reflected similar themes to the composite of the same name mentioned above: valuing interpersonal relationships and in-person interactions. “*Positive Self-Focus*” reflected themes related to a sudden recognition that something is important, valuing mental health/mood state, and becoming truer to oneself via introspection. “*Wellness Self-Management”* reflected a focus on engaging in activities aimed at improving health and wellness and making family and its long-term continuation a top priority. “*Primacy of Health*” reflected themes related to a pandemic-induced renewed appreciation for small things and specific health-related concerns.

Who’s Important was summarized by four composite scores that explained 59% of the variance. “*Primacy of Interpersonal Concerns*” reflected themes related to prioritizing interpersonal relationships. “*Isolation & Disengagement*” reflected themes related to dealing with quarantine restrictions and letting go of people or activities as a result. “*Epiphanic Clarity*” reflected a new realization of the importance of something or intensified feelings about something always recognized as important, and mental health concerns. “*Family Welfare*” reflected themes related to the long-term priority of family well-being and a de-emphasis on the seriousness of things.

Focus of Life Energy was summarized by seven composite scores that explained 52% of the variance. “*Active in the World*” reflected themes related to travel, long-term family well-being, and interpersonal relationships. “*True to Self*” reflected themes related to prioritizing becoming truer to oneself and introspection. “*Hobbies*” reflected themes related to engaging in leisure activities and creative pursuits. “*Seriousness*” reflected similar ideas to above, that is a new realization or intensified feelings of the importance of something and taking things more seriously. “*Wellness Self-Management*” reflected themes related to activities of health maintenance and focusing on health issues.

### Latent profiles

**Figure 3:**
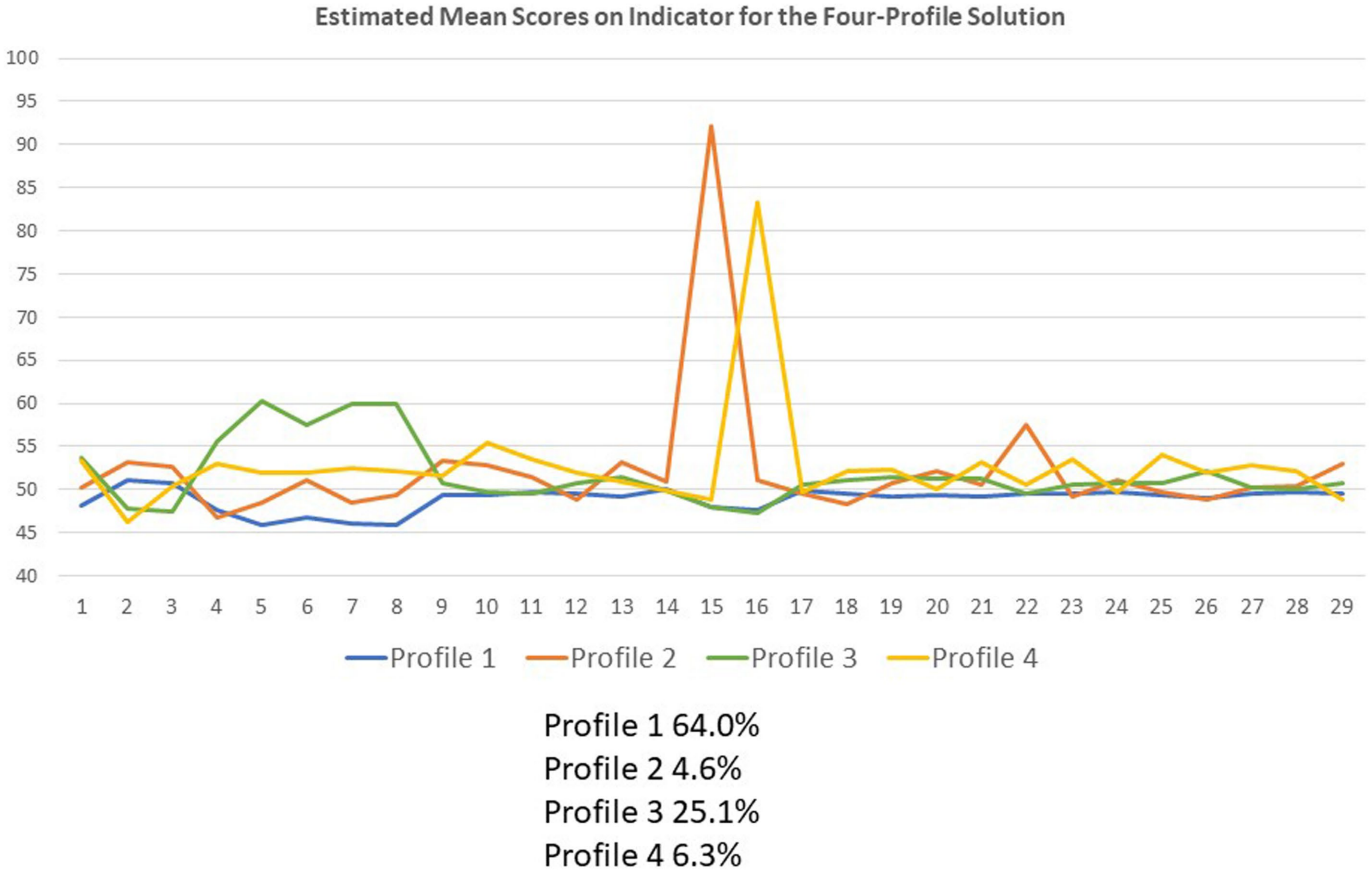
Plot of mean scores on the 29 indicators by profile. This line graph shows the plot of the mean scores for each of the 29 indicators by profile. The indicator content is shown in Table 2.

Five LPA models were tested (one-to five-profile solutions tested) using 29 indicators derived from the factor analyses and principal components analysis. The four-profile model fit the data best, showing the lowest AIC and BIC and the highest entropy. The significant LRT indicated that the four-profile model improved fit over the three-profile model (*p* = 0.03; Supplementary Table S7). Table 2 shows the factor-and composite-score means on the 29 indicators, conditionally formatted to highlight the magnitude and direction of the scores, with greater magnitude reflected by more saturated color. Red highlighting reflected scores lower than the *T*-score means of 50, whereas green highlighting reflected scores higher than the *T*-score means of 50. Figure 3 shows the plot of the mean scores for each of the 29 indicators by profile. The link between the indicator number and its content is shown in Table 2. Conditional formatting indicates the ES based on Cohen’s d for a T-score metric, with increased color saturation indicating larger ES and the direction of the difference from a mean score of 50 shown in pink hues for scores below 50 and in green hues for scores above 50.

**Table 2:** Factor and composite score means for four-profile lpa solution.^a^

Indicator #	Variable	Profile 1	Profile 2	Profile 3	Profile 4
		(*n* = 772)	(*n* = 55)	(*n* = 295)	(*n* = 75)
		Index Group	COVID-19-specific health & resignation to isolation attributable to COVID-19	High stress, low trust	Active in the world, low trust
1	Perceived change—inner life—self and relationships *T*-score	48.2	50.3	53.7	53.3
2	Norms—public health confidence and consideration *T*-score	51.0	53.2	47.7	46.3
3	Norms—trust in leaders and media *T*-score	50.7	52.7	47.5	50.4
4	Norms—Public Incivility *T*-score	47.7	46.8	55.6	53.0
5	Stress—quality of life *T*-score	45.9	48.5	60.2	52.0
6	Stress—racism/inequity *T*-score	46.8	51.1	57.5	52.0
7	Stress—financial hardship *T*-score	46.0	48.4	59.9	52.5
8	Stress—family relationships *T*-score	46.0	49.4	59.9	52.2
9	Meaning—surviving COVID-19 *T*-score	49.3	53.3	50.7	51.5
10	Meaning—post-traumatic growth *T*-score	49.4	52.8	49.7	55.4
11	Meaning—interpersonal connections *T*-score	49.8	51.4	49.5	53.5
12	Meaning—renewed appreciation *T*-score	49.6	48.9	50.8	51.9
13	Meaning—health concerns *T*-score	49.1	53.1	51.4	50.9
14	Meaning—circumstances *T*-score	50.0	50.9	49.8	49.9
15	What is important—COVID prevention *T*-score	47.9	92.1	48.0	48.8
16	What is important—primacy of employment *T*-score	47.7	51.1	47.3	83.4
17	What is important—interpersonal connections *T*-score	49.9	49.6	50.5	49.7
18	What is important—positive self-focus *T*-score	49.5	48.3	51.0	52.2
19	What is important—wellness self-management *T*-score	49.2	50.7	51.4	52.3
20	What is important—primacy of health *T*-score	49.3	52.2	51.3	50.0
21	Who is important—primacy of interpersonal connections *T*-score	49.2	50.6	51.2	53.2
22	Who is important—isolation and disengagement *T*-score	49.5	57.5	49.6	50.7
23	Who is important—epiphanic clarity *T*-score	49.5	49.3	50.6	53.5
24	Who is important—family welfare *T*-score	49.7	51.0	50.7	49.8
25	Focus on—active in the world *T*-score	49.3	49.8	50.8	54.1
26	Focus on—true to self *T*-score	49.1	48.9	52.1	51.9
27	Focus on—hobbies *T*-score	49.6	50.3	50.3	52.9
28	Focus on—seriousness *T*-score	49.8	50.3	50.0	52.1
29	Focus on—wellness self-management *T*-score	49.6	53.0	50.8	48.8

a All scores are on a *T*-score metric, with sample-specific mean of 50, and standard deviation of 10. 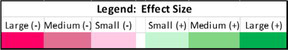

Individuals in Profile 1—named “*Index Group*”—included 64% of the sample and had relatively average scores on all indicators although they had scores reflecting a lower endorsement of perceived public incivility, stress related to racism/inequity and financial hardship, the importance of COVID-19 prevention, and the primacy of employment (all small ES).

Those in Profile 2—named “*COVID-Specific Health & Resignation to Isolation Attributable to COVID*”—included 5% of the sample, and their scores suggested a particular focus on COVID-specific preventive health behaviors along with noting the requisite isolation and disengagement entailed in the social distancing necessary for COVID-19 prevention (large and medium ES, respectively). They had scores reflecting a greater sense of public-health confidence/consideration, and a greater trust in leaders/media. Their changing definition of the meaning of QOL comprised actions taken to survive COVID-19, endeavors to enable post-traumatic growth, and multiple health-and wellness-related concerns (all small ES). Thus, these individuals believe in the public-health messages and are doing the things they need to do to take precautions and protect their health. The consequences of this perspective are that they feel resigned to being isolated and lonely, and they do not see a way out.

Profile 3—named “*High Stress, Low Trust*”—included 25% of the sample and had high multi-domain stress (three of the four were large ES), high perceived public incivility, perceived change toward inner life/self and relationships, and true to self (medium, small and small ES, respectively). They had low trust/confidence in others’ public health practices and in the leadership/media, in the primacy of employment, and the importance of COVID-19 prevention (all small ES). This group is under stress in every domain of their life but does not trust the public health messages or put much emphasis on the importance of COVID-19 prevention compared to others. They are the group with the most elevated scores both on focusing on being true to themselves and in perceiving people to be increasingly uncivil.

Profile 4—named “*Active in the World, Low Trust*”—included 6% of the sample and placed a particular emphasis on employment (large ES) and other activities (small ES), and emphasized multiple aspects of interpersonal relationships, personal growth, and introspection (medium and small ES). They also endorsed higher levels of perceived public incivility and reported stress in health-related QOL, financial hardship, and family relationships (small ES). Individuals in this group were focusing on trying to get back into the world, especially work. They feel that the pandemic has helped them to put their priorities in focus and are trying to find more meaning in their life activities.

### Demographic differences by latent profile

Table 3 shows the sociodemographic characteristics of the four profile groups, and the results of chi-squared or ANOVA analyses comparing groups, for categorical and continuous variables, respectively. The profiles were different on 10 of the 20 variables compared. On the categorical variables, the profile groups differed on race, marital status, difficulty paying bills, employment status, number of times they reported having had COVID-19, number of COVID-19 boosters received, and whether they had Long COVID (all small ES). On the continuous variables, the profile groups differed in age, BMI, and number of comorbidities (medium, small, and small ES, respectively).

**Table 3:** Overall and profile-group demographic characteristics.

Variable		Profile group characteristics								Profile differences	
		Profile 1 (*n* = 772)		Profile 2 (*n* = 55)		Profile 3 (*n* = 295)		Profile 4 (*n* = 75)		Cramer’s *V*	
		Index group		COVID-19-specific health & resignation to isolation attributable to COVID-19		High stress, low trust		Active in the world, low trust			
		#	%	#	%	#	%	#	%		
Role	Patient	506	66%	38	69%	195	66%	52	69%	0.08	
	Caregiver	103	13%	3	5%	50	17%	16	21%		
	Both	29	4%	0	0%	16	5%	2	3%		
	Neither	134	17%	14	25%	34	12%	5	7%		
	Missing	0	0%	0	0%	0	0%	0	0%		
Gender	Male	141	18%	8	15%	35	12%	6	8%	0.09	
	Female	629	81%	47	85%	257	87%	68	91%		
	Other	2	0%	0	0%	3	1%	1	1%		
	Prefer not to answer	0	0%	0	0%	0	0%	0	0%		
Race	White	719	93%	51	93%	248	84%	69	92%	**0.10**	
	Person of color/multiracial	43	6%	4	7%	32	11%	2	3%		
	Prefer not to answer	10	1%	0	0%	15	5%	4	5%		
Living alone	Yes, living alone	125	16%	10	18%	37	13%	10	13%	0.05	
Marital status	Never married	98	13%	2	4%	46	16%	13	17%	**0.11**	
	Married	466	60%	35	64%	156	53%	44	59%		
	Cohabitation/Domestic	35	5%	1	2%	25	8%	4	5%		
	Separated	7	1%	0	0%	8	3%	0	0%		
	Divorced	88	11%	14	25%	47	16%	9	12%		
	Widowed	75	10%	3	5%	11	4%	5	7%		
	Prefer not to answer	3	0%	0	0%	2	1%	0	0%		
Difficulty paying bills	Not at all difficult	525	68%	35	64%	71	24%	42	56%	**0.25**	
	Slightly difficult	149	19%	11	20%	77	26%	18	24%		
	Moderately difficult	45	6%	4	7%	76	26%	6	8%		
	Very difficult	31	4%	3	5%	34	12%	3	4%		
	Extremely difficult	12	2%	1	2%	32	11%	5	7%		
	Not applicable/Prefer not to answer	10	1%	1	2%	5	2%	1	1%		
Employment status	Employed	268	35%	16	29%	143	48%	53	71%	**0.20**	
	Unemployed	49	6%	4	7%	29	10%	3	4%		
	Retired	303	39%	21	38%	24	8%	13	17%		
	Medically disabled	140	18%	14	25%	96	33%	6	8%		
	Do not know/Prefer not to answer	12	2%	0	0%	3	1%	0	0%		
Education (at baseline)	Less than high school graduate	3	0%	1	2%	2	1%	0	0%	0.06	
	High school diploma/GED	64	8%	5	9%	24	8%	4	5%		
	Trade or technical degree	46	6%	2	4%	25	8%	3	4%		
	Some college	172	22%	11	20%	68	23%	15	20%		
	College degree	240	31%	20	36%	95	32%	19	25%		
	Postgraduate degree	246	32%	15	27%	81	27%	33	44%		
	Missing	1	0%	1	2%	0	0%	1	1%		
Region (at baseline)	East North Central	106	14%	5	9%	55	19%	13	17%	0.10	
	East South Central	29	4%	2	4%	14	5%	3	4%		
	Middle Atlantic	82	11%	4	7%	44	15%	5	7%		
	Mountain	73	9%	5	9%	24	8%	9	12%		
	New England	44	6%	3	5%	15	5%	5	7%		
	Other US or International	53	7%	3	5%	18	6%	2	3%		
	Pacific	118	15%	13	24%	39	13%	19	25%		
	South Atlantic	181	23%	18	33%	57	19%	10	13%		
	West North Central	43	6%	0	0%	13	4%	3	4%		
	West South Central	43	6%	2	4%	16	5%	6	8%		
Currently smoke or vape	Not at all	700	91%	50	91%	256	87%	70	93%	0.05	
	Some days	22	3%	2	4%	11	4%	2	3%		
	Every day	44	6%	2	4%	27	9%	3	4%		
	Prefer not to answer	6	1%	1	2%	1	0%	0	0%		
Received help completing survey	Yes	5	1%	1	2%	6	2%	1	1%	0.06	
Number of times has had COVID-19	0	353	46%	31	56%	104	35%	31	41%	**0.15**	
	1	313	41%	17	31%	119	40%	33	44%		
	2	77	10%	5	9%	43	15%	8	11%		
	3	12	2%	0	0%	19	6%	1	1%		
	4	2	0%	0	0%	4	1%	0	0%		
	5	0	0%	2	4%	0	0%	0	0%		
	Other	8	1%	0	0%	5	2%	1	1%		
	Do not remember	5	1%	0	0%	0	0%	1	1%		
	Missing	2	0%	0	0%	1	0%	0	0%		
Received a COVID-19 vaccine	No	67	9%	5	9%	39	13%	3	4%	0.08	
	Yes	692	90%	49	89%	254	86%	71	95%		
	Do not remember	3	0%	1	2%	1	0%	0	0%		
	Missing	10	1%	0	0%	1	0%	1	1%		
Received 1+ COVID-19 booster	No	143	19%	7	13%	63	21%	5	7%	0.09	
	Yes	605	78%	47	85%	228	77%	68	91%		
	Do not remember	1	0%	1	2%	0	0%	0	0%		
	Missing	23	3%	0	0%	4	1%	2	3%		
Number of COVID-19 boosters (if received 1 or more boosters)	1	78	10%	6	11%	43	15%	15	20%	**0.11**	
	2	184	24%	16	29%	68	23%	16	21%		
	3	223	29%	15	27%	49	17%	18	24%		
	Other	41	5%	2	4%	13	4%	6	8%		
	Do not remember	2	0%	0	0%	0	0%	0	0%		
	Missing	77	10%	8	15%	55	19%	13	17%		
Have Long COVID	Definitely not	438	57%	39	71%	89	30%	35	47%	**0.19**	
	Probably not	134	17%	6	11%	58	20%	16	21%		
	Probably yes	42	5%	0	0%	49	17%	9	12%		
	Definitely yes	23	3%	1	2%	33	11%	2	3%		
	Do not know	65	8%	7	13%	38	13%	6	8%		
	Missing	70	9%	2	4%	28	9%	7	9%		
		**Mn**	**SD**	**Mn**	**SD**	**Mn**	**SD**	**Mn**	**SD**	**Eta** ^**2**^	**Significant post-hoc pairwise differences (Scheffe)**
Age		59.91	13.23	61.64	11.78	49.89	11.50	53.64	11.935	0.11	1 vs. 3, 1 vs. 4, 2 vs. 3, 2 vs. 4
	Missing	0		0		0		0			
Body mass index (at baseline)		28.68	6.96	30.47	8.79	31.40	9.31	29.81	7.53	0.02	1 vs. 3
	Missing	23		2		10		4			
Comorbidities		3.71	2.45	4.11	2.65	4.99	2.63	4.23	2.52	0.04	1 vs. 3
	Missing	2		0		1		0			
Time since diagnosis (diagnosis date reported, at baseline)		18.20	13.09	19.2	12.68	17.53	11.46	17.01	11.56	0.00	n/a
	Missing	29		5		6		2			

GED, General Educational Development (i.e., high-school equivalency test); SD, standard deviation; Mn, mean; SD, standard deviation. “Prefer not to respond,” “do not remember,” “do not know,” “not applicable,” and “other” responses were excluded from the chi-square test. The bolded values of Cramer’s V are all small effect sizes.

**Figure 4:**
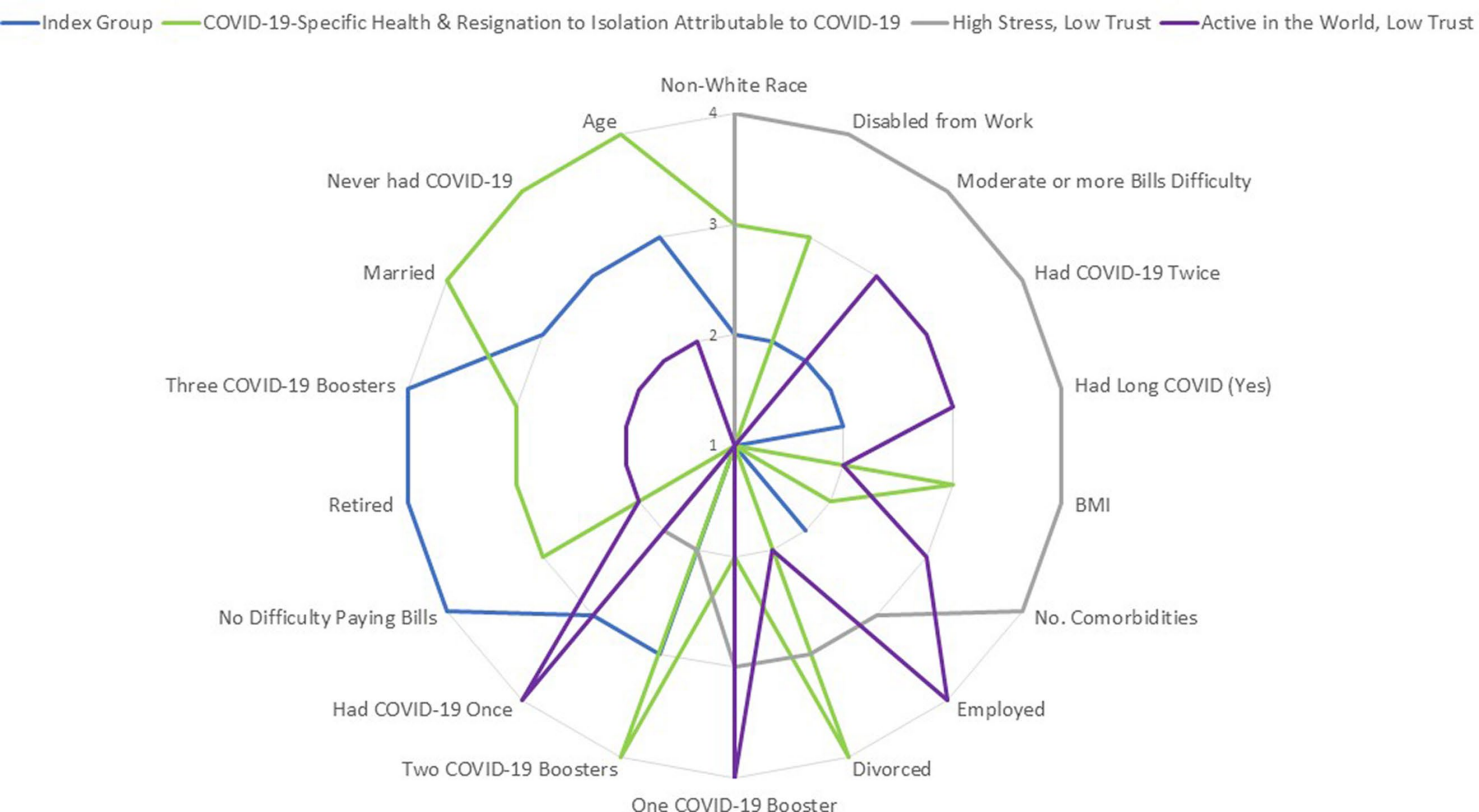
Radar chart of prominent characteristics by profile group. This radar chart summarizes the four profile-group differences in the sociodemographic variables compared. Only variables with at least a small ES difference are shown. Higher ranks reflect having a higher proportion with, or scores on, this characteristic. For ease of comparison, the ranks shown in this figure were sorted first by Profile 3 and then by Profile 1, so that pertinent characteristics were grouped together on the radar plot.

Figure 4 summarizes these profile differences using a radar plot of ranks for each variable with at least a small ES difference. Higher ranks reflect having a higher proportion with, or scores on, this characteristic. For ease of comparison, the ranks shown in this figure were sorted first by Profile 3 and then by Profile 1, so that pertinent characteristics were grouped together on the radar plot.

Profile 1 (Index Group) reflects a group that is largely retired, with no reported difficulty paying bills, and which had three COVID-19 boosters. Profile 2 (COVID-19-Specific Health & Resignation to Isolation Attributable to COVID) stands out by dint of having the highest age, never having had COVID-19, having the highest proportion of married participants and of divorced participants, and having had two COVID-19 boosters. In contrast, Profile 3 (High Stress, Low Trust) reflects a group that has the highest proportion of non-White individuals, who reported moderate or worse difficulty paying bills, had a disproportionate number of individuals disabled from work due to a medical condition, had had COVID-19 twice, and reported having had Long COVID. They also reported the highest number of comorbidities and the highest BMI. Profile 4 (Active in the World, Low Trust) stands out by dint of having the largest proportion of employed individuals, who had had one COVID-19 booster and had had COVID-19 once.

## Discussion

Three years after the beginning of the worldwide COVID-19 pandemic, its impact is notable on most study participants’ conceptualization of QOL, their priorities, perspectives on social norms, and perceived stressors. About 75% of the study sample noted changes in these aspects of life, and overall reported the greater prominence of family and friends in their values. Similar to findings from early in the pandemic, a focus on family welfare (e.g., concern about child development, caring for others) was prominent. But many of the concerns highlighted early in the pandemic were not mentioned in our data, such as prioritizing order, stability, and conformity (49, 52).

Overall, participants also reported the importance of health and its protection and noted that the pandemic made them take things more seriously. It gave participants a renewed appreciation for what had been the “small things,” such as spending time in person with those they love, appreciating medical care providers, or valuing “the abundance of ordinary life… such as the ability to have family and friends close, receive health care as needed, and having food and entertainment always available” (exemplary direct quote).

While the present study was not longitudinal, its findings do reflect concepts consistent with response-shift phenomena (75, 76) by dint of the nature of the questions and prompts asked. Similar to many other studies that have utilized cross-sectional qualitative data to learn about ways that people’s values, priorities, and concerns do and do not change (77–79), such work may have implications for greater insight into the nature of QOL appraisal and response shift. The present study revealed that in response to the catalyst of the pandemic, people perceived changes in how they thought about QOL and either identified new priorities or had epiphanies about their values. Many perceived a change in how they preferred to spend their time, valuing alone time, solitary pursuits, and unscheduled time. As the world continues to change in response to the changing conditions of COVID-19, one wonders whether these perceived changes in priorities and values will persist.

Given the backdrop of these overall trends, further analysis revealed that the sample could be further characterized as comprising four profiles. In comparison to the Index group, which was the largest group and generally had average scores on the 29 indicators of perceived change, the other three profile groups differed in their reactions to COVID-19 restrictions and ways of coping. Individuals in Profile 2, the smallest group, adhered closely to COVID-19 restrictions and adapted to a more selective social world to maintain social distancing and preserve their health as they saw fit. In contrast, individuals in Profile 3, the second largest group, were less focused on COVID-19-specific preventions in their open-response comments, possibly due to a lack of trust in guidelines recommended by public-health officials, elected leaders, and the media. Of note, they expressed that people were more uncivil than before the pandemic, and their experience of multi-domain stressors was much higher than people in the other profile groups. Due to this high stress, there may have been more immediate topics on their minds than COVID-19 prevention at the time of the survey. Instead, individuals in Profile 3 focused on being true to themselves and attending to their inner life. Although individuals in Profile 4 shared with Profile 3 a distrust of public health officials and elevated reported public incivility and multi-domain stress, they appeared to cope in more adaptive ways. This group prioritized their work, personal growth, and wellness self-management.

The four profile groups differed in demographic characteristics as well, with the participants of Profile 3 comprised of more individuals of non-white race (11% of the group as compared to 3–7% of the other profile groups), who reported worse financial difficulties, higher BMI and more comorbidities, and more individuals who were disabled from work due to a medical condition. Of note, they were more likely to have had COVID-19 more than once and to report having Long COVID. Despite Profile 4 individuals sharing with Profile 3 a distrust of public health officials and a focus on their inner life, they had a lower COVID-19 burden and were more likely than any of the other groups to be employed. Profile 2 individuals reported greater focus on adhering to COVID-19-guidelines and were the oldest in the sample, reported never having had COVID-19, had multiple booster vaccinations, and were more likely to be married or divorced. Individuals in Profile 3 may have had fewer economic resources to access public health.

Our study findings thus revealed that in the face of the global pandemic, individuals dealt with the accompanying stress and despair in psychologically distinct ways. A small minority of the sample engaged in strong adherence to COVID-19 restrictions, but they also endured consequential isolation and disengagement. Two groups shared a distrust of public-health leaders, but one seemed to suffer the most negative consequences both in terms of multi-domain stress and Long COVID. The other group focused their attention on their work and were more buffered from the negative consequences.

While the present research did not address the efficacy of treatments to improve participants’ mental health, recent research has noted evolutions in mental health systems of care in response to the acute concerns during COVID-19 (80). This evolution focused on infection control, continuity of care for mental-health service users, and facilitating remote access to mental-health assessment and care in the context of new-onset or high-risk patients (80). Such interventions were facilitated by time efficiency and flexibility, but often failed to reach specific vulnerable populations and those with low technological literacy (81). Future research might examine whether remote treatments impact individuals’ priorities and concerns, possibly using similar methods and measures as used in the present work.

### Limitations

The present study has notable advantages in its use of both qualitative and quantitative data. Its substantial sample size also enabled a careful series of data reduction and multivariate analyses, and the resulting profiles made theoretical sense. The study limitations should be noted, however. First, the attrition from baseline is notable and both the baseline participation rate and the causes for this attrition remain unknown. While it could be due to the usual reasons hindering survey research (e.g., lack of interest or time), it is also possible that it is due in part to COVID-19-related mortality. The selection bias analyses implicated only three characteristics in the attrition out of 16 considered, and two of these may reflect social determinants of health (more financial difficulties and lower education). The study sample is also less representative of non-white and/or Hispanic individuals, so the generalizability of study findings to these race/ethnicity groups is limited (80). Its generalizability to other countries, cultures, and healthcare systems is also unknown and may also be limited. The attrition and data may also reflect other biases (82). Social desirability may also play into participant responses to an unknown extent, in that they might have limited their disclosures of perceived changes due to their own theories about what can and cannot be said. Finally, the qualitative data used in this study is based on open-ended data from an online survey, researchers have no control of the depth of material provided by study participants and therefore relevant information might have been missed. Future research might build on the current findings by examining how health and well-being outcomes differed by profile group. Such research might also examine the experience of Long COVID, and how social problems such as domestic violence (83) relate to the detected changes in priorities and concerns. Other researchers might build on the current work by utilizing the same set of questions in other, more diverse samples.

## Conclusion

In summary, the present study revealed that participants perceived substantial changes in priorities and/or values due to the COVID-19 pandemic in three-quarters of the sample. The four profile groups identified reflected distinct ways of dealing with COVID-19-prevention guidelines, some adapting by adherence and resignation, some by increasingly focusing on inner life and others by balancing engagement in the world with a focus on inner life. Future research might examine the impact of these different coping approaches on health and well-being outcomes.

## Data availability statement

The datasets presented in this article are not readily available because the study data are confidential and thus not able to be shared. Requests to access the datasets should be directed to CS, carolyn.schwartz@deltaquest.org.

## Ethics statement

The studies involving humans were approved by WCG Independent Review Board (#2021164). The studies were conducted in accordance with the local legislation and institutional requirements. The participants provided their written informed consent to participate in this study.

## Author contributions

CS: Conceptualization, Data curation, Formal analysis, Methodology, Project administration, Supervision, Validation, Visualization, Writing – original draft, Writing – review & editing. KB: Formal analysis, Methodology, Validation, Writing – review & editing. AW: Formal analysis, Writing – review & editing. TS: Formal analysis, Writing – review & editing. BC: Formal analysis, Writing – review & editing. EA: Formal analysis, Writing – review & editing. I-CH: Funding acquisition, Writing – review & editing. GR: Funding acquisition, Writing – review & editing. BR: Conceptualization, Writing – review & editing. RS: Formal analysis, Funding acquisition, Writing – review & editing.

## Footnotes

^1^www.rarepatientvoice.com

^2^www.ipsos.com

^3^www.alchemer.com
