# Supplementary material for: Emerging priorities and concerns in the wake of the COVID-19 pandemic: qualitative and quantitative findings from a United States national survey
Source: Front Public Health. 2024 Jun 19;12:1365657. doi: 10.3389/fpubh.2024.1365657 (PMC11221197; doi:10.3389/fpubh.2024.1365657)
Supplement: Supplementary file 1 [file Table_1.pdf]

Supplementary Table 1. Selection Bias Comparisons on Demographic Characteristics

Variable		Baseline Sample (n=4756)		Selection Bias Comparison: Retained Sample (n=1197)		Not Retained (n=3559)		Cramer's V
		#	%	#	%	#	%	
Role	Patient	3044	64%	791	66%	2253	63%	0.03
	Caregiver	675	14%	172	14%	503	14%	
	Both	186	4%	47	4%	139	4%	
	Neither	851	18%	187	16%	664	19%	
	Missing	0	0%	0	0%	0	0%	
Gender	Male	849	18%	190	16%	659	19%	0.03
	Female	3,879	82%	1,001	84%	2878	81%	
	Other	23	0%	6	1%	17	0%	
	Prefer not to answer	4	0%	0	0%	4	0%	
	Missing	1	0%	0	0%	1	0%	
Race	White	4125	87%	1087	91%	3038	85%	0.07
	Person of color/multiracial	502	11%	81	7%	421	12%	
	Prefer not to answer	128	3%	29	2%	99	3%	
	Missing	1	0%	0	0%	1	0%	
Living Alone	Yes, living alone	576	12%	160	13%	416	12%	0.02
	Unchecked	4,179	88%	1,037	87%	3142	88%	
	Missing	1	0%	0	0%	1	0%	
Marital Status	Never Married	770	16%	172	14%	598	17%	0.07
	Married	2,644	56%	732	61%	1912	54%	
	Cohabitation/Domestic	323	7%	72	6%	251	7%	
	Separated	90	2%	14	1%	76	2%	
	Divorced	667	14%	142	12%	525	15%	
	Widowed	239	5%	64	5%	175	5%	
	Missing	23	0%	1	0%	22	1%	
Difficulty Paying Bills	Not at all Difficult	2292	48%	672	56%	1620	46%	**0.10**
	Slightly Difficult	1073	23%	250	21%	823	23%	
	Moderately Difficult	699	15%	137	11%	562	16%	
	Very Difficult	321	7%	70	6%	251	7%	
	Extremely Difficult	260	5%	43	4%	217	6%	
	Not applicable/Prefer not to answer	110	2%	25	2%	85	2%	
	Missing	1	0%	0	0%	1	0%	
Employment Status	Employed	1957	41%	471	39%	1486	42%	0.09
	Unemployed	594	12%	132	11%	462	13%	
	Retired	913	19%	306	26%	607	17%	
	Medically Disabled	1220	26%	276	23%	944	27%	
	Missing	72	2%	12	1%	60	2%	
Education (at Baseline)	Less than high school graduate	55	1%	6	1%	49	1%	**0.10**
	High school diploma/GED	457	10%	97	8%	360	10%	
	Trade or technical degree	308	6%	76	6%	232	7%	
	Some college	1292	27%	266	22%	1026	29%	
	College degree	1407	30%	374	31%	1033	29%	
	Postgraduate degree	1222	26%	375	31%	847	24%	
	Missing	15	0%	3	0%	12	0%	
Region	East North Central	751	16%	179	15%	572	16%	0.070
	East South Central	222	5%	48	4%	174	5%	
	Middle Atlantic	571	12%	135	11%	436	12%	
	Mountain	436	9%	111	9%	325	9%	

Variable		Baseline Sample (n=4756)		Selection Bias Comparison				
				Retained Sample (n=1197)		Not Retained (n=3559)		Cramer's V
	New England	226	5%	67	6%	159	4%	
	Other US or International	305	6%	76	6%	229	6%	
	Pacific	601	13%	189	16%	412	12%	
	South Atlantic	1066	22%	266	22%	800	22%	
	West North Central	239	5%	59	5%	180	5%	
	West South Central	339	7%	67	6%	272	8%	
Currently Smoke or Vape	Not at all	3939	83%	1062	89%	2877	81%	0.09
	Some days	243	5%	37	3%	206	6%	
	Every day	538	11%	92	8%	446	13%	
	Missing	36	1%	6	1%	30	1%	
Received Help Completing Survey	Yes	77	2%	14	1%	63	2%	0.02
	No	4678	98%	1,183	99%	3495	98%	
	Missing	1	0%	0	0%	1	0%	
COVID-19 Infection Status	Yes	375	8%	84		291	8%	0.02
		**Mn**	**SD**	**Mn**	**SD**	**Mn**	**SD**	**Cohen's d**
Age		51.58	14.18	54.13	13.418	50.72	14.33	**-0.24**
	Missing	1		0		1		
Body Mass Index (at baseline)		29.98	8.29	29.50	7.80	30.14	8.44	0.08
	Missing	208		39		169		
Comorbidities		3.66	2.41	3.64	2.34	3.66	2.44	0.01
	Missing	10		0		10		
Time Since Diagnosis (diagnosis date reported at baseline)		14.66	12.57	15.00	12.583	14.55	12.56	-0.04
	Missing	279		42		237		

GED = General Educational Development (i.e., high-school equivalency test)

Mn= mean; SD = standard deviation

"Prefer not to respond," "do not know" and "not applicable" responses were excluded from the chi-square test.

Supplemental Table 2: Description of Selected Themes Used in Coding Open-Text Data

No.	QOL Definition Themes	Definitions	Verbatim Examples of Associated Language
1	Balance	Balancing different priorities, work/life balance	I am more focused on relationships than tasks. Less work focus- better life balance.
2	Circumstances	Longer term situations	living situation, safety, financial issues, employment change
3	Contentment	A current or desired feeling state of living with enjoyment, fulfillment, feeling of well-being, happiness, satisfaction	Enjoy.., grateful for…., living life to the fullest, holistic well-being, appreciate
4	Contribution	Make a difference to larger society (beyond family)	Be able to volunteer and participate
5	COVID-19 Accommodations Retained	Continuing to engage in preventive behaviors to reduce exposure to COVID-19 (e.g., wearing mask, social distancing).	I have for the most part resumed my pre-COVID-19 activities but drive less now and have masks with me but only use them occasionally. I do keep socially distant whenever possible.
6	Educational Pursuit	Going back to school	Online classes, registering in new degree program
7	Family / friends	Anything specific to family	Interact with family members,
8	Gravity	Taking things more seriously, pandemic-induced renewed appreciation for something	Appreciate small things more, new level of importance
9	Health	Feeling physically and/or emotionally healthy	Health, healthy,
10	In-person	Valuing in-person interactions	Not taking for granting time spent with ____,
11	Independence	Wanting to be able to perform normal activities with no assistance or as little as possible	Do what I want when I want, do it by myself; do without worry
12	Minimizing COVID-19 Risk	Reducing risk of contracting risk	Much more concerned about my surroundings and protecting my health.
	No Change	No change on matter of prompt	No change.
13	No Direct Answer	no answer at all	Blank, N/A, no response, My quality of life is ok" or "Yes", decline to answer, too broad to answer

14 Personal Growth	Belief that one's life is purposeful and meaningful and a sense of continued growth and development as a person. Ability to change or take positive action in life circumstances.	Feeling enlightened
15 Physical Touch	Appreciating the unique value of being touched by others.	Made me realize that I appreciate hugs a lot more.
16 Positive Attitude	An attitude of optimism about expectations and manner of interpreting life. Judging life positively. Intentionally positive about prospect of health, energy, happiness.	One day at a time, being happy with what I have, noticing improvement, in a good frame of mind
17 Problems	Shorter-term – Dealing with some specific issue. While pain is a problem to live with, it is bigger and more pervasive	Difficulty with, need help w/..., have car problem, have child care problem
18 Provider-related	Something about MD or facility	difficulty reaching MD
19 Reminiscence	thinking back, wistfulness	get help with my back problems so I can start doing some of the things that I did before I started having these problems; fear and sorrow about how pain is increasing over what it was
20 Survival	Mortality	Not wanting to jump off the balcony would be nice; this pain is sucking the life out of me.
21 Treatment-related	Comments related to their pathology - physical complaints, diagnoses, symptoms, treatments, meds	Neuropathy, stenosis, pain, broken foot, immobility
22 Wellness Lifestyle	Engaging in activities to promote or protect health	I've adopted a healthier lifestyle in order to enjoy better quality of life

No.	Who is important, What is important, Life-Energy Focus Question Themes	Definitions	Verbatim Examples of Associated Language
1	Acceptance	Feeling okay with what one has. Needs to be stated in positive, i.e. do not code non-acceptance as acceptance	To become more happy with what I have; to be as good as possible for my age; accept my rate of recovery, no longer embarrassed to wear a mask
2	Accomplishing Chores & Tasks	Able to do normal, day to day activities	Take care of myself, keep my house up, cooking, shopping, walk the dog
3	Achievements	Aspires to specific big and distal personal goal. Needs to be stated in the positive, i.e. do not code this for non-achievement or pain free	Able to do long-distance bike trips, set up and run a new and successful business, build a family, finish [writing] my book
4	Bubble: Family	COVID-19-related isolation made me choose family to include in my bubble	Spend time visiting family during pandemic-induced isolation
5	Bubble: Friends	COVID-19-related isolation made me choose friends to include in my bubble	Spend time outdoors with friends during pandemic-induced isolation
6	Community Involvement & Volunteering	Social involvement beyond family; more specific, focused contributions than in "Social & Altruistic Concerns"	Volunteer at a soup kitchen, return to coaching minor hockey, "Continue to be a source of inspiration to my students and instill them with self-confidence."
7	COVID-19-Related Misinformation / Conspi	References COVID-19-related fallacies	COVID-19 was planned, anti-vax ideas, anti-asian racism related to transmission
8	COVID-19-Specific	Specific desires related to the COVID-19-19 Pandemic	I want COVID-19 over, I want to be able to not wear a mask again, I want the COVID-19 vaccine
9	Creating moments & memories	Prioritization of quality time, doing things had postponed but wanted to do	Living in the moment, spending time with ___, taking loved ones out, I want to back to activities with others
10	Creative activities	Activities such art, sewing, knitting, etc.	i've had to put more energy into my home space - art, gardening, hobbies
11	Disengagement	Letting go of life, people, activities anticipating death from age or illness (not as in mood disorder). Similar to Existential but without the emotion.	I've had to cut off relationships with some people who said they cared for me but wouldn't do simple things to keep me safe in a pandemic.

12	Drug & Alcohol Use	Drug and alcohol overuse, dependency or addiction	I have weaned myself off of 12 months of oxycodone and Gabapenten but not back to normal sleep.
13	Education	Education	perhaps do a master’s degree, learn Hebrew
14	Environmental Welfare	General environmental wellbeing, sustainability oriented	Slow global warming; reduce melting of the ice caps; stop climate change
15	Epiphanic clarity	Relating to a moment where suddenly realize something as important	intensified my feelings about the things I held as important before, no time for drama, appreciating life, understanding what truly want to do not what others expect, realizing the value that we can create ourselves, valuing more the abundance of ordinary life
16	Essential Self	Becoming more myself	More introspective, reflecting on and reflecting more of who I truly am, being happy in solitude
17	Existential Concerns	Explicit mention of purpose in life, concerned with one’s fate and lot in life. NOT based on Sartre-esque existence precedes meaning philosophy	Frame of mind that what if there is no remedy; Helplessness; Dissatisfaction with life; I do not want to accept that my good years are all behind me; Health issues that have been inflicted upon me; Feeling frustrated because of lack of accomplishments; Nothing is the same; Do not want to be a burden ; Loss of appetite for experiencing things; Give up hope; Accept this will never be fully resolved; Avoid sinking into depression.
18	Family Welfare	Benefit of subject’s family, individually oriented, family is top priority	Be there for my grandchildren; have children; Raise my children well, my family is most (more) important
19	Fantasy	Completely impossible goals	Be 20 years younger; Wake up one morning and everything would be 100% back to the way it was; Have super strength and laser vision; Bigger Penis

20	Financial Concerns	Specific to money. Code Work goal for jobs and retirement. Specific concerns about themselves, not general financial concerns about society	Financial freedom; Provide for my family
21	Financial Welfare (Societal):	Financial concerns as with financial welfare, specifically related to money, general societal concerns about money and not individual concerns.	I wish people weren't poor; I wish people had enough money to afford a house and food everyday; I wish there was no debt
22	Gravity	Taking things more seriously, pandemic-induced renewed appreciation for something	Appreciate small things more, new level of importance
23	Grieving COVID-19 loss(es)	Mentions loved one(s) who died of COVID-19, grieving the loss of what used to be	I lost my cousin due to COVID-19 so it makes it even more real just how deadly this can be.
24	Health & Wellness	Mentions good health, caring about wellness	I still value my good health
25	Health Issues	Mentions specific physical, cognitive, or emotional complaints, problems, treatments. Not necessarily spine-related. For ability to think clearly, memory, cognitive, code as Health + Mental Health. Includes exercise for fitness or for a specific health problem; otherwise code exercise as Leisure.	Includes all kinds of pain, physical and emotional conditions, treatments. E.g. need to lose weight or exercise; difficulty with strength, balance, sleep, ability to sit, stand, walk, be active, be mobile. Physical fitness.
26	Health Welfare (Societal)	Desires related to specific health on a general scale, not related to specific individuals	Cure for cancer; No more broken bones; No one born with incurable disease; Profound amount of suffering that's been experienced in abundance by so many.
27	Immigration & Citizenship	Any concerns related to citizenship status	It's hard getting treatment without being a citizen
28	In-person	Valuing in-person interactions	Not taking for granting time spent with ____,
29	Independent Functioning	Wants to be able to perform activities with no assistance or as little as possible Expressed as such; don't infer.	Do what I want when I want, do it by myself; do without worry, able to do normal …; able to continue driving; Look after myself

30	Interpersonal Relationships	Meaningful ongoing interaction and feeling of connection with family or others	Retire with my wife, babysit my grandchildren, help my aging parents, socialize with friends, friends top priority
31	Isolation	Dealing with quarantine restrictions	It was hard to be told that visiting them was not a good idea. Realizing that I didn't have anyone in my inner circle due to quarantine.
32	Legacy	Concerned with future of family, cultivating future of family	I want to stay healthy so I can be here to see my grandchildren have children.
33	Legal & Crime (Societal)	Legal and crime issues related to general societal trends and standings, not directly related to the individual or close persons	Murder rates to go down; Drugs to be legalized
34	Legal & Crime / Safety Concerns	Legal, crime, and safety issues directly related to the individual or close persons	Fear of falling; avoid high risk activities; concerned about daughter in danger at school getting in with bad kids
35	Leisure Activities	Ability and opportunity to do things that bring pleasure. Does not include retirement. Code Leisure for "being active" or "doing activities". Could also include items under Tasks if framed as pleasant and optional.	Physically active life; mention hiking, skiing, golfing, boating, fishing, hunting, gardening, playing
36	Living Situation (Societal)	Housing and living for general peoples in society, not specifically related to the individual	End homelessness; Everyone has a roof over their head; Everyone feels safe when at home
37	Living Situation, Housing, Neighborhood	Goal for your living situation	Want to move to be near my daughter; proceed with renovations, stay in my home as long as possible; nursing home; downsize; like to live alone; home-bound, improving my home space
38	Maintenance	Keep, not lose, maintain, continue, stay, remain	To remain physically and mentally agile; keep what I have now; not get any worse
39	Material Acquisitions (less)	Less focused on obtaining material possessions.	[The pandemic] made us appreciate that we don't need 'stuff' for the kids or us.
40	Material Acquisitions (more)	More focused on obtaining material possessions; specifically not financial gain.	I want a _____, to get a bunch of ________, etc. Specific reference to object

41 Mental Health & Mood State	Emotional and mental states. Includes cognitive issues. Includes enjoyment	Enjoy life; Retain my positive outlook, Decluttering my home to declutter my mind, Be happy, not worry, work/life balance; Memory; Mental confusion; Stress (mental); grateful or appreciate or fortunate that I did not lose loved ones in the pandemic
42 No Change	No change on matter of prompt	No change.
43 No Direct Answer	Did not give any answer. Also, “Everything” or “Nothing / None” is equivalent to Not Applicable N/A	N/A, blank, no response, decline to answer, answers only with a question to clarify; Not sure; Too broad; silly question
44 Political Welfare	Specific mention of political issues and government, if general issue is related to politics do not code	A better president; Redrawn districts; Quicker legal process; Get rid of certain politicians
45 Prevention	Prevent, avoid,	Strengthen my whole body to support my back better, Lose weight to take extra stress off my back/body; Prevent my back from going out; prevent further damage
46 Prioritization	Changing priorities due to pandemic	I began to examine all activities and weigh the risks vs benefits, prioritizing family and friends and moments.
47 Problem Resolution	Deals with some specific shorter-term issues. Pain is a problem to live with but this theme is not related to physical symptoms unless stated as such	need help w/… , fix or replace car that breaks down a lot, find child care, get household help,.
48 Provider & Treatment-Related Concerns	Specific concerns about MD et al or medical interventions. Not necessarily physical or spinal. Includes equipment	I would like to have an expert tell me if my medications are correct; Less visits to the doctor. Wheel chair; cane; hospitalization; surgery; medications; physiotherapy
49 Racism	Specific to concerns about race and racism, including concern about race-specific risk factors, health disparities, and experiences with providers.	Everyone should have equal opportunities

50 Regret / Missed Opportunities	Feeling regretful at missed opportunities in one's life due to focusing on different priorities.	...allowed me to see the importance of home. I've been a flight attendant for 37 years and I missed out on so many things being away all the time. Family. I missed opportunities
51 Religious & Spiritual Concerns	Religious & Spiritual Concerns	Enlightenment, holistic well-being
52 Self-image & Personality	how you and others see you, how you want or don't want to be	Maintain my self-dignity, "not embarrassed", Being a loving wife, mother, grandmother, sister, aunt, etc.
53 Social & Altruistic Concerns	Big ideas; broader caring about or providing for others, philanthropy, advocacy	"making a difference", do random acts of kindness; wanting future success for grandchildren
54 Travel	Desire or ability to go some distance to visit places or people	Want to travel
55 Work & Unemployment	Job-related. Includes anything about retirement. Code Financial Concerns for money-related.	Want to retire, be able to work more than 5 hours; do profession-related writing, research & teaching; find a job and work as a normal person, get a job where I am appreciated.

Supplemental Table 3. Inter-rater reliability for open-ended prompts (6 raters, 41 rows, 246 raters*rows)

	Total Marked Across All Raters	Percent Marked Out of Total Rows x Raters	Kappa
**Q1 Meaning**			
Q1 Code. No Direct Answer	70	28%	0.569
Q1 Code. No change	58	24%	0.910
Q1 Code. Family / friends	41	17%	0.713
Q1 Code. Health	26	11%	0.381
Q1 Code. Minimizing COVID-19 risk	35	14%	0.687
Average			**0.652**
**Q2 What's Important**			
Q2 Code. No Direct Answer	30	12%	0.651
Q2 Code. No change	45	18%	0.831
Q2 Code. Family Welfare	50	20%	0.628
Q2 Code. Health & Wellness	27	11%	0.476
Q2 Code. Interpersonal Relationships	50	20%	0.518
Average			**0.621**
**Q3 Who's Important**			
Q3 Code. No Direct Answer	67	27%	0.799
Q3 Code. No change	100	41%	0.879
Q3 Code. Family Welfare	42	17%	0.506
Q3 Code. Interpersonal Relationships	39	16%	0.421
Average			**0.651**
**Q4 Focus On**			
Q4 Code. No Direct Answer	42	17%	0.724
Q4 Code. Creating Moments & Memories	24	10%	0.280
Q4 Code. Epiphanic clarity	30	12%	0.423
Q4 Code. Health and Wellness	42	17%	0.610
Q4 Code. Interpersonal Relationships	36	15%	0.557
Q4 Code. Mental Health and Mood State	48	20%	0.669
Average			**0.544**

Percent Marked Out of Total Rows x Raters >=15%

Percent Marked Out of Total Rows x Raters >=10% BUT <15%

Supplemental Table 4: Prevalence of Themes by Prompt in Open-Text Data

No.	QOL Definition Themes	% Endorsement in COVID-19 Resilience FU3 data
		QOL Meaning Prompt
1	Balance	2.9%
2	Circumstances	4.4%
3	Contentment	7.4%
4	Contribution	0.7%
5	COVID-19 Accommodations Retained	6.3%
6	Educational Pursuit	0.5%
7	Family / friends	15.6%
8	Gravity	13.2%
9	Health	15.1%
10	In-person	5.9%
11	Independence	3.8%
12	Minimizing COVID-19 Risk	14.5%
	No Change	24.4%
13	No Direct Answer	22.8%
14	Personal Growth	9.2%
15	Physical Touch	0.3%
16	Positive Attitude	9.4%
17	Problems	3.5%
18	Provider-related	0.7%
19	Reminiscence	1.7%
20	Survival	5.3%
21	Treatment-related	1.8%
22	Wellness Lifestyle	2.2%

Supplemental Table (contd): Prevalence of Themes by Prompt in Open-Text Data

	**Who is important, What is important, Life-Energy Focus Question Themes**	*What is Important Prompt*	*Whot is Important Prompt*	*Life Energy Focus Prompt*
1	Acceptance	1.2%	0.3%	0.4%
2	Accomplishing Chores & Tasks	0.5%	0.0%	0.8%
3	Achievements	0.3%	0.2%	0.9%
4	Bubble: Family	1.0%	1.6%	0.6%
5	Bubble: Friends	0.2%	0.7%	0.1%
6	Community Involvement & Volunteering	1.3%	0.5%	3.6%
7	COVID-19-Related Misinformation / Conspiracy Theory	1.2%	1.8%	0.9%
8	COVID-19-Specific	2.7%	2.0%	1.3%
9	Creating moments & memories	3.6%	1.7%	6.3%
10	Creative activities	0.4%	0.2%	2.3%
11	Disengagement	1.9%	2.1%	2.1%
12	Drug & Alcohol Use	0.1%	0.0%	0.0%
13	Education	0.3%	0.1%	1.3%
14	Environmental Welfare	0.3%	0.0%	0.3%
15	Epiphanic clarity	13.0%	5.9%	10.0%
16	Essential Self	3.1%	1.3%	4.9%
17	Existential Concerns	1.3%	0.1%	1.2%
18	Family Welfare	19.0%	21.8%	8.9%
19	Fantasy	0.0%	0.0%	0.2%
20	Financial Concerns	0.3%	0.1%	0.8%
21	Financial Welfare (Societal):	0.1%	0.3%	0.1%
22	Gravity	5.8%	4.3%	3.2%
23	Grieving COVID-19 loss(es)	0.8%	1.3%	0.5%
24	Health & Wellness	12.8%	1.9%	8.6%
25	Health Issues	4.6%	1.1%	2.9%
26	Health Welfare (Societal)	1.0%	0.7%	0.5%
27	Immigration & Citizenship	0.2%	0.0%	0.2%
28	In-person	2.6%	1.1%	0.8%
29	Independent Functioning	1.6%	0.2%	1.2%
30	Interpersonal Relationships	19.6%	30.0%	11.9%
31	Isolation	1.7%	2.5%	0.2%
32	Legacy	0.7%	0.2%	1.3%
33	Legal & Crime (Societal)	0.0%	0.0%	0.4%
34	Legal & Crime / Safety Concerns	0.0%	0.0%	0.0%
35	Leisure Activities	1.2%	0.2%	0.0%
36	Living Situation (Societal)	0.0%	0.0%	4.0%
37	Living Situation, Housing, Neighborhood	1.5%	0.2%	0.0%
38	Maintenance	0.8%	0.1%	0.8%
39	Material Acquisitions (less)	1.8%	0.3%	0.3%
40	Material Acquisitions (more)	0.2%	0.0%	1.3%

Supplemental Table (contd): Prevalence of Themes by Prompt in Open-Text Data

	**Who is important, What is important, Life-Energy Focus Question Themes**	*What is Important Prompt*	*Whot is Important Prompt*	*Life Energy Focus Prompt*
41	Mental Health & Mood State	5.8%	2.7%	0.1%
42	No Change	22.6%	32.0%	8.1%
43	No Direct Answer	19.3%	24.1%	26.1%
44	Political Welfare	1.5%	0.5%	23.9%
45	Prevention	3.0%	1.8%	0.8%
46	Prioritization	5.3%	3.3%	1.3%
47	Problem Resolution	0.2%	0.0%	4.3%
48	Provider & Treatment-Related Concerns	0.7%	0.5%	0.3%
49	Racism	0.0%	0.0%	0.3%
50	Regret / Missed Opportunities	0.2%	0.3%	0.0%
51	Religious & Spiritual Concerns	2.1%	1.2%	0.1%
52	Self-image & Personality	1.0%	0.8%	1.3%
53	Social & Altruistic Concerns	1.5%	0.9%	1.8%
54	Travel	1.6%	0.2%	1.6%
55	Work & Unemployment	2.7%	0.8%	2.7%

Supplemental Table 5. Results of Factor Analyses By Domain

Domain: Perspective Changes

Item	Factor Loading: Inner Life & Relationships	Factor Loading: Job	Total Explained Variance
Having free time has become more important to me	**0.851**		
Having time for myself has become more important to me	**0.746**		
Solving problems in my relationships with my family and friends has become more important to me	**0.642**		
Keeping up relationships with my family and friends has become more important to me	**0.602**		
My job has become more important to me		**0.746**	
Where I work (e g , the organization, its mission or goal) has become more important to me		**0.734**	
Eigenvalue	2.618	1.463	
Variance Explained	36.029	17.328	53.357
Alpha reliability	0.79	0.72	

Domain: Social Norms

Item	Factor Loading<br>Public Health Confidence & Consideration	Trust in Leaders & Media	Public Incivility	Total Explained Variance
I am confident that others will wear a mask when they are sick	**0.794**			
I am confident that others have gotten vaccinated against COVID-19	**0.707**			
The general public would do what is necessary to protect vulnerable populations if there were a future pandemic	**0.528**			
Elected leaders have the best interests of the general public in mind when making COVID-19 related policy decisions		**0.778**		
The media is providing accurate information about COVID-19		**0.743**		
I have experienced or people losing their temper in public (e g , road rage)			**0.693**	
I have experienced more people being less polite or considerate of strangers			**0.685**	
Eigenvalue	2.328	1.379	1.329	
Variance Explained	26.531	13.792	11.700	52.023
Alpha reliability	0.73	0.75	0.64	

Domain: Stress

Item	Health-Related Quality of Life Stress	Systemic Racism/Inequity	Financial Hardship	Family Relationship Stress	Total Explained Variance
	Factor Loading				
Your physical health stress	**0.696**		0.271		
Getting proper medical care stress	**0.642**		0.253		
Your social life, social activities, friendships stress	**0.609**			0.382	
Your mental health stress	**0.596**		0.392	0.263	
The COVID-19-19 pandemic stress	**0.555**				
Using public services (e.g., Social Services, health clinics) stress	**0.515**	0.301			
Transportation (e.g., driving, traffic, commuting) stress	0.460	0.331			
Serious injury, illness, or death of someone close to you stress	0.457				
Relation with ethnic/racial groups other than your own stress		**0.771**			
Relations with police (e.g., harassment, availability) stress	0.252	**0.613**			
Experiences involving racism/discrimination stress		**0.607**			
Crime and violence (e.g., physical assault, robbery, murder) stress	0.229	**0.563**			
Your neighborhood environment (e g , safety, cleanliness, noise, pollution, graffiti) stress	0.359	0.451			
Your education (e g , college, training program) stress		0.284		0.235	
Alcohol or drugs (e..g, use of alcohol or drugs in self or others) stress		0.270		0.234	
Money or finances stress	0.248		**0.769**		
Housing, your living situation stress	0.307		**0.639**		
Your job situation (e g job experiences, unemployment, career satisfaction) stress			**0.516**	0.329	
Raising children/being a parent/problems with children stress				**0.561**	
Marriage, romantic relationships stress	0.357		0.283	**0.550**	
Caring for elderly parents or other relatives stress	0.272			0.335	
Eigenvalue	6.74	1.82	1.37	1.18	
Variance Explained	29.56	6.34	3.92	2.86	42.682
Alpha reliability	0.84	0.75	0.74	0.58	

Supplemental Table 6. Results of Principal Components Analysis by Prompt

Prompt: QOL Meaning

Item	Component Loading						Total Explained Variance
	**Surviving COVID-19**	**Post-traumatic growth**	**Interpersonal connections**	**Renewed appreciation**	**Health concerns**	**Circumstances**	
Meaning - Minimizing COVID-19 risk	**0.778**						
Meaning - COVID-19 accommodations retained	**0.676**						
Meaning - Survival	**0.613**			0.208			
Meaning - Personal Growth		**0.707**					
Meaning - Positive Attitude (mental health)		**0.698**		0.228		0.216	
Meaning - Balance		**0.566**					
Meaning - Family / friends			**0.814**				
Meaning - In Person			**0.809**				
Meaning - Gravity				**0.797**			
Meaning - Contentment				**0.649**			
Meaning - Problems					**0.769**	0.240	
Meaning - Health	0.227				**0.704**	-0.330	
Meaning - Wellness Lifestyle					0.208	**-0.666**	
Meaning - Circumstances					0.247	**0.638**	
Eigenvalue	1.68	1.50	1.35	1.23	1.11	1.02	
Variance Explained	12.00	10.69	9.63	8.76	7.92	7.30	56.29

Prompt: What's Important

	Component Loading						Total Explained Variance
	**COVID-19 Prevention**	**Primacy of Employment**	**Interpersonal connections**	**Positive self-focus**	**Wellness self-management**	**Primacy of Health**	
What's Important - Prevention	**0.808**						
What's Important - COVID-19-Specific	**0.801**						
What's Important - Prioritization		**0.764**					
What's Important - Work and Unemployment		**0.752**					
What's Important - Interpersonal Relationships			**0.722**		0.240		
What's Important - In Person			**0.719**				
What's Important - Epiphanic clarity		0.286		**0.694**			
What's Important - Mental Health and Mood State				**0.608**			
What's Important - Essential Self			-0.332	**0.540**			
What's Important - Health and Wellness					**0.717**		
What's Important - Family Welfare		0.214			**0.673**		
What's Important - Gravity						**0.790**	
What's Important - Health Issues	0.242					**0.633**	
Eigenvalue	1.51	1.42	1.24	1.16	1.09	1.07	
Variance Explained	11.64	10.90	9.57	8.89	8.42	8.26	57.68

Prompt: Who's Important

	Component Loading				
	**Primacy of Interpersonal Concerns**	**Isolation & Disengagement**	**Epiphanic Clarity**	**Family Welfare**	**Total Explained Variance**
Who's Important - Interpersonal Relationships	**0.703**				
Who's Important - Prioritization	**0.564**				
Who's Important - Isolation		**0.768**			
Who's Important - Disengagement		**0.759**			
Who's Important - Mental Health and Mood State			**0.814**		
Who's Important - Epiphanic clarity	0.300		**0.666**		
Who's Important - Family Welfare	0.327			**0.769**	
Who's Important - Gravity	0.466			**-0.644**	
Eigenvalue	1.416	1.202	1.070	1.018	
Variance Explained	17.706	15.030	13.374	12.721	58.83

Prompt: Focus of Life Energy

	Component Loading						
	**Active in the World**	**True to Self**	**Hobbies**	**Seriousness**	**Wellness self-management**		**Total Explained Variance**
Focus On - Interpersonal Relationships	**0.588**						
Focus On - Family Welfare	**0.631**						
Focus On - Travel	**0.632**						
Focus On - Essential Self		**0.731**					
Focus On - Prioritization		**0.510**	-0.307				
Focus On - Leisure Activities			**0.723**				
Focus On - Creative Activities			**0.584**				
Focus On - Gravity				**0.852**			
Focus On - Epiphanic clarity		**0.535**		**0.557**			
Focus On - Health and Wellness			0.238		**0.736**		
Focus On - Health Issues		-0.204	-0.225		**0.706**		
Eigenvalue	1.38	1.34	1.14	1.08	1.05		
Variance Explained	12.56	12.142	10.339	9.782	9.528	54.35	

** Dropped because uninterpretable*

Supplemental Table 7. Summary of Latent Profile Model Fit							
**No. Profiles**	**No. Free Parameters**	**AIC**	**BIC**	**Sample Adj BIC**	**Entropy**	**Smallest Class Probability (Est Posterior Prob)**	**P-value for LO-MENDELL-RUBIN ADJUSTED LRT TEST**
1 profile	58	256,596.73	256,891.80	256,707.57			
2 profile	88	255,293.50	255,741.20	255,461.68	0.82	0.29	0.00
3 profile	118	253,479.71	254,080.04	253,705.23	0.89	0.05	0.00
4 profile	148	252,384.58	253,137.54	252,667.44	0.92	0.05	0.03
5 profile	*Model not identified due to non-positive definite first-order derivative product matrix.*						
