# Supplementary material for: Emerging priorities and concerns in the wake of the COVID-19 pandemic: qualitative and quantitative findings from a United States national survey
Source: Front Public Health. 2024 Jun 19;12:1365657. doi: 10.3389/fpubh.2024.1365657 (PMC11221197; doi:10.3389/fpubh.2024.1365657)
Supplement: Supplementary file 2 [file Data_Sheet_1.PDF]

Supplemental Text:
COVID-Follow-Up 3 Items for Mixed Methods Appendix

Perspective Changes

1. When you think back on your experiences with the COVID pandemic, how have you changed how you think about the meaning of **quality of life** for you?

2. When you think back on your experiences with the COVID pandemic, how have you changed how you think about **what is important in your life**?

3. When you think back on your experiences with the COVID pandemic, how have you changed how you think about **who is important in your life**?

4. When you think back on your experiences with the COVID pandemic, how have you changed how you think about **what you want to focus on or spend your life energy on**?

Changes in Priorities

5. My job has become more important to me.

- Strongly agree
- Moderately agree
- Neutral
- Moderately disagree
- Strongly disagree
- Not applicable

6. Where I work (e.g., the organization, its mission or goal) has become more important to me.

- Strongly agree
- Moderately agree
- Neutral
- Moderately disagree
- Strongly disagree
- Not applicable

7. Keeping up relationships with my family and friends has become more important to me.

- Strongly agree
- Moderately agree
- Neutral
- Moderately disagree
- Strongly disagree
- Not applicable

8. Solving problems in my relationships with my family and friends has become more important to me.

- Strongly agree
- Moderately agree
- Neutral
- Moderately disagree
- Strongly disagree
- Not applicable

9. Having time for myself has become more important to me.

- Strongly agree
- Moderately agree
- Neutral
- Moderately disagree
- Strongly disagree
- Not applicable

10. Having free time has become more important to me.

- Strongly agree
- Moderately agree
- Neutral
- Moderately disagree
- Strongly disagree
- Not applicable

11. I am willing to take a pay cut so that I can work from home.

- Strongly agree
- Moderately agree
- Neutral
- Moderately disagree
- Strongly disagree
- Not applicable

Social Norms

12. I am confident that others will wear a mask when they are sick.

- Strongly agree
- Moderately agree
- Neutral
- Moderately disagree
- Strongly disagree
- Not applicable

13. I am confident that others have gotten vaccinated against COVID.

- Strongly agree
- Moderately agree
- Neutral
- Moderately disagree
- Strongly disagree
- Not applicable

14. I have experienced more people being less polite or considerate of strangers.

- Strongly agree
- Moderately agree
- Neutral
- Moderately disagree
- Strongly disagree
- Not applicable

15. Elected leaders have the best interests of the general public in mind when making COVID related policy decisions.

- Strongly agree
- Moderately agree
- Neutral
- Moderately disagree
- Strongly disagree
- Not applicable

16. The media is providing accurate information about COVID.

- Strongly agree
- Moderately agree
- Neutral
- Moderately disagree
- Strongly disagree
- Not applicable

17. The general public would do what is necessary to protect vulnerable populations if there were a future pandemic.

- Strongly agree
- Moderately agree
- Neutral
- Moderately disagree
- Strongly disagree
- Not applicable

18. I have experienced more people losing their temper in public (e.g., "road rage").

- Strongly agree
- Moderately agree
- Neutral
- Moderately disagree
- Strongly disagree
- Not applicable

Life Stress COVID

19. ***Please indicate how much stress you have experienced related to each statement below.***

	Extreme Stress	A Lot of Stress	Some Stress	Little Stress	No Stress	Do not know / Not applicable	Decline to answer
Money or finances *	○	○	○	○	○	○	○
Housing, your living situation *	○	○	○	○	○	○	○
Your job situation							

(e.g. job experiences, unemployment, career satisfaction) *							
Your education (e.g., college, training program) *							
Your neighborhood environment (e.g., safety, cleanliness, noise, pollution, graffiti) *							
Using public services (e.g. Social Services, health clinics) *							
Transportation (e.g. driving, traffic, commuting) *							
Alcohol or drugs (e.g. use of alcohol or drugs in self or others) *							
Crime and violence (e.g. physical assault, robbery, murder) *							
Relations with police (e.g. harassment, availability) *							
Relations with ethnic/racial groups other than your own *							
Experiences involving racism/discrimination *							
Your physical health *							
Your mental health							
Getting proper medical care *							

medical care *							
Serious injury, illness or death of someone close to you *							
Raising children/being a parent/problems with children *							
Marriage, romantic relationships *							
Your social life, social activities, friendships *							
Are there any other areas of your life that are stressful (like immigration, sexual abuse): Please specify.							
The COVID-19 pandemic							
Caring for elderly parents or other relatives							
Enter another opti							
