# Supplementary figures and images for: Emerging priorities and concerns in the wake of the COVID-19 pandemic: qualitative and quantitative findings from a United States national survey
Source: Front Public Health. 2024 Jun 19;12:1365657. doi: 10.3389/fpubh.2024.1365657 (PMC11221197; doi:10.3389/fpubh.2024.1365657)

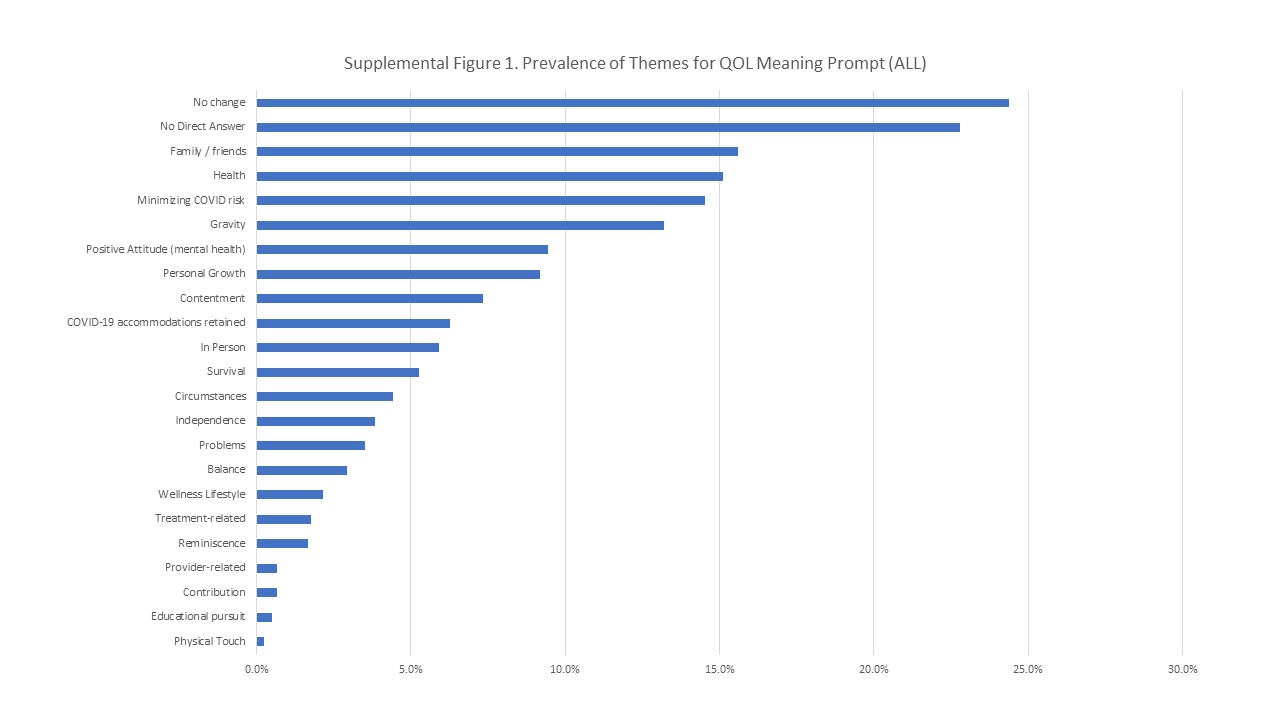

Supplement: Supplementary file 3 [file Image_1.JPEG]

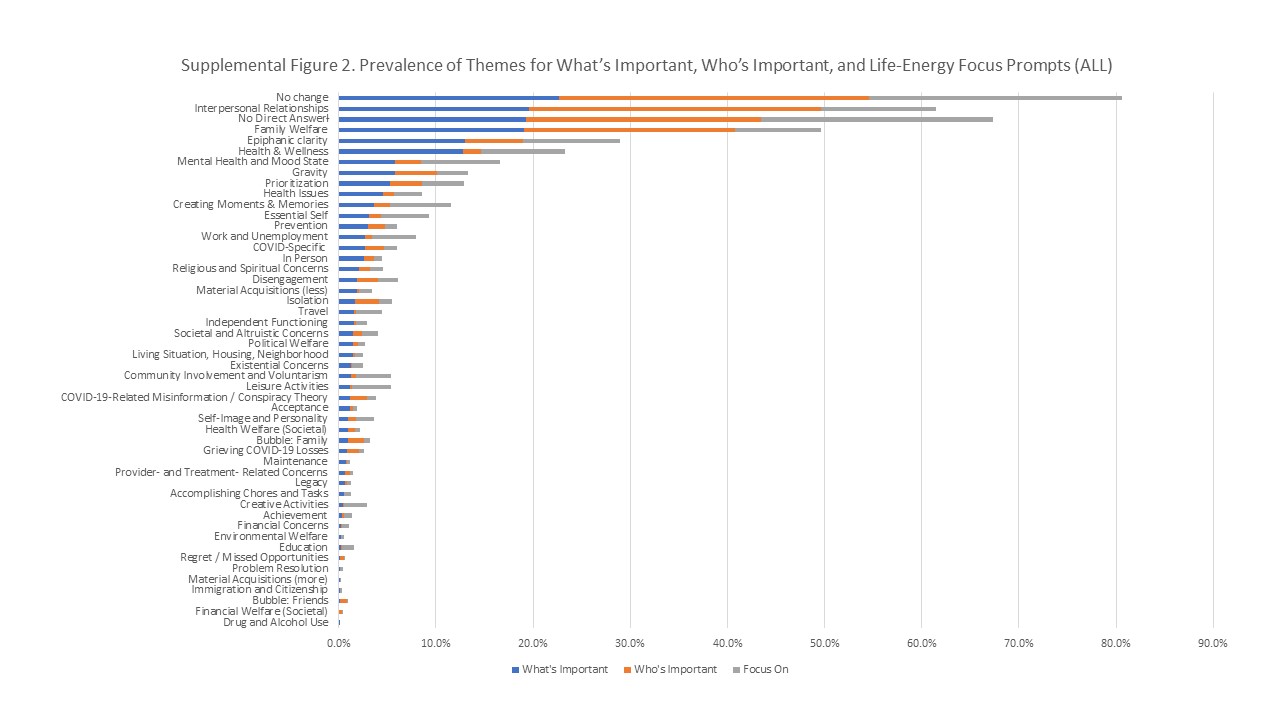

Supplement: Supplementary file 4 [file Image_2.JPEG]
